# Onco-Cardiology: Consensus Paper of the German Cardiac Society, the German Society for Pediatric Cardiology and Congenital Heart Defects and the German Society for Hematology and Medical Oncology

**DOI:** 10.1007/s00392-020-01636-7

**Published:** 2020-05-13

**Authors:** Tienush Rassaf, Matthias Totzeck, Johannes Backs, Carsten Bokemeyer, Michael Hallek, Denise Hilfiker-Kleiner, Andreas Hochhaus, Diana Lüftner, Oliver J. Müller, Ulrich Neudorf, Roman Pfister, Stephan von Haehling, Lorenz H. Lehmann, Johann Bauersachs, Committee for Clinical Cardiovascular Medicine of the German Cardiac Society

**Affiliations:** 1 Department of Cardiology and Vascular Medicine, West German Heart and Vascular Centre Essen, University Hospital Essen, Hufelandstrasse 55, 45147 Essen, Germany; 2 Institute for Experimental Cardiology, University Hospital Heidelberg, Heidelberg, Germany; 3 Department of Oncology, Hematology and Bone Marrow Transplantation with the Section Pneumology, Centre for Oncology, University Hospital Hamburg-Eppendorf, Hamburg, Germany; 4 Department I of Internal Medicine, Center for Integrated Oncology ABCD, University Hospital of Cologne, Cologne, Germany; 5 Department of Cardiology and Angiology, Hannover Medical School, Hannover, Germany; 6 Department of Hematology and Medical Oncology, University Hospital Jena, Jena, Germany; 7 Department of Haematology, Oncology and Tumour Immunology, Charité, Humboldt University Berlin, Berlin, Germany; 8 Department of Internal Medicine III (Cardiology, Angiology and Internal Intensive Care Medicine), University Hospital Schleswig-Holstein, University of Kiel, Kiel, Germany; 9 Department of Pediatrics III, West German Heart and Vascular Centre Essen, University Hospital Essen, Essen, Germany; 10 Clinic III for Internal Medicine, General and Interventional Cardiology, Electrophysiology, Angiology, Pneumology and Internal Intensive Care Medicine, University Hospital Cologne, Cologne, Germany; 11 Department of Cardiology and Pneumology, Heart Center Göttingen, University of Göttingen Medical Center and German Center for Cardiovascular Research (DZHK), partner site Göttingen, Göttingen, Germany; 12 Department of Cardiology, Angiology, Pneumology, University Hospital Heidelberg, Heidelberg, Germany

**Keywords:** Cardio-oncology, Cardiotoxicity, Survivorship programs, Cancer therapy, Chemotherapy

## Abstract

The acute and long-lasting side effects of modern multimodal tumour therapy significantly impair quality of life and survival of patients afflicted with malignancies. The key components of this therapy include radiotherapy, conventional chemotherapy, immunotherapy and targeted therapies. In addition to established tumour therapy strategies, up to 30 new therapies are approved each year with only incompletely characterised side effects. This consensus paper discusses the risk factors that contribute to the development of a potentially adverse reaction to tumour therapy and, in addition, defines specific side effect profiles for different treatment groups. The focus is on novel therapeutics and recommendations for the surveillance and treatment of specific patient groups.

## Cardiovascular risk factors and diseases—risk assessment prior to tumour therapy

**Fig. 1:**
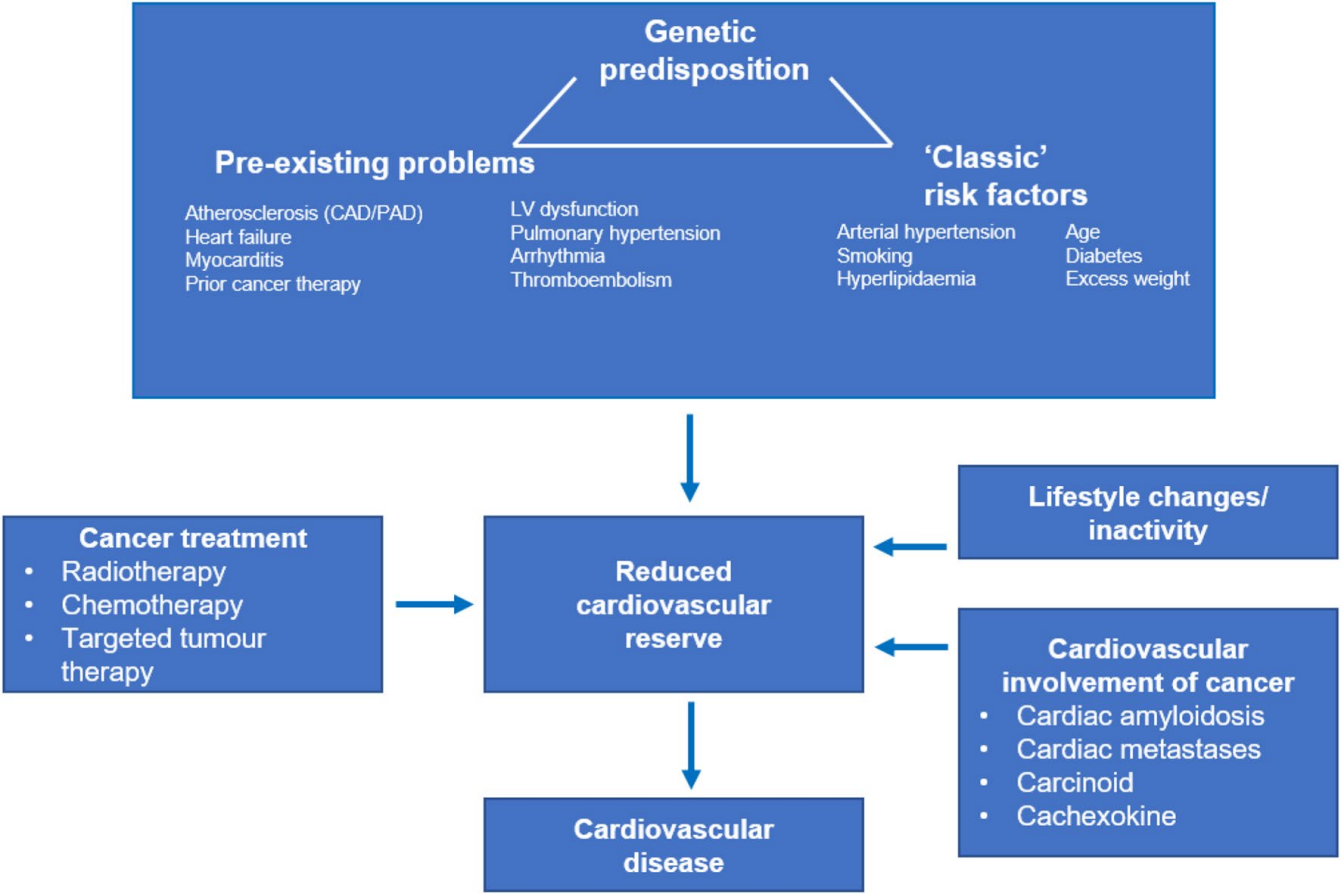
Interaction of genetic predisposition, classic risk factors and pre-existing cardiovascular diseases in the development or progression of a cardiovascular disease as a result of cancer therapy (‘2nd hit’) or the cancer itself. *CAD* coronary artery disease,* PAD* peripheral artery disease, *LV* left ventricular

Many oncology patients have pre-existing risk factors or cardiovascular diseases, which can lead to a wide range of cardiovascular complications as a consequence of the cancer and, in particular, cancer therapies. Furthermore, the incidence of side effects has been found to be impacted both by a genetic predisposition and by the influence of specific cancer-derived factors, which remain inadequately characterised (Fig. 1) [11, 57, 99, 149]. Cardiovascular consequences range from discrete alterations in electrocardiography, laboratory tests or imaging to the occurrence of thromboembolism, ischaemic or arrhythmic events, left ventricular (LV) dysfunction and heart failure [140, 149]. Therefore, it is imperative for clinicians to record pre-existing cardiovascular diseases by thoroughly evaluating the medical history and to evaluate cardiovascular conditions prior to any oncological therapy. In addition to physical examination, the electrocardiogram (ECG) and echocardiogram, including qualitative and quantitative assessment of the LV ejection fraction (LVEF), play a central role, and increasingly also cardiac magnetic resonance imaging (MRI). In the case of subclinical or manifest cardiac pathology, an alternative, less cardiotoxic treatment may be considered, or close monitoring may be required for early detection of cardiovascular alterations during cancer therapy, which may then enable cardioprotective therapy.

The significance of classic risk factors, pre-existing and concomitant cardiovascular diseases and previous cancer therapies is discussed below.

### Classic risk factors

Cardiovascular disease and cancer not only share common mechanisms such as chronic inflammation but also exhibit common risk factors [47, 90]. Diabetes mellitus, obesity and hypercholesterolaemia are not only considered to be risk factors for breast cancer but also are associated with increased cardiovascular side effects [67, 101]. Although the reduced cardiotoxicity observed in a retrospective study of breast cancer patients treated with statins needs to be confirmed in prospective studies [17], we consider the treatment of hypercholesterolaemia to be useful. By analogy, diabetes mellitus should also be treated according to guidelines. Where possible, metformin should also be part of the treatment, as epidemiological studies suggest a reduced cancer rate in diabetics treated with metformin [44]. Smoking, the classic risk factor for cancer, is also associated with an increased risk for atherosclerosis and coronary artery disease (CAD) [90] and deserves specific consideration. The significance of pre-existing CAD for acute coronary events associated with specific medications such as 5-fluorouracil (5-FU) remains to be conclusively established [1, 121].

High blood pressure is also associated with an increased cancer rate, at least in men, and with increased cancer mortality in both sexes [132]. Indeed, inhibitors of the renin–angiotensin–aldosterone system were associated with a lower incidence of metastasis and improved survival in cancer patients [135]. Therefore, it is important to identify and treat arterial hypertension both before starting cancer therapy and during the course of treatment.

The relevance of these shared risk factors is also apparent in patients who are scheduled to undergo anthracycline therapy and who are at increased risk of developing heart failure in the presence of pre-existing risk factors such as smoking, arterial hypertension, diabetes mellitus and dyslipidemia [5]. In the presence of more than two concomitant risk factors, these patients’ risk appears to increase significantly [5–7]. Among classic cardiac risk factors, there is also evidence of an increased cardiovascular risk for patients after stem cell transplant [24] as well as patients treated with specific tyrosine kinase inhibitors such as ponatinib or nilotinib, which supports the prognostic benefits of risk factor monitoring [18].

### Pre-existing or concomitant cardiovascular diseases

The identification of pre-existing or concomitant cardiovascular diseases is central to risk assessment. In addition to medical history, a physical examination helps to identify not only heart failure but also atherosclerotic manifestations such as peripheral arterial occlusive disease, which can be further complicated by some kinase inhibitors [88].

Identifying ECG anomalies and any current or prior cardiac arrhythmias is an integral part of cardiac risk assessment. Specific medications can cause potentially harmful prolongation of the frequency-corrected QT interval (QTc) on a 12-lead ECG. Although potentially life-threatening arrhythmias with a precedent of QTc interval prolongation (e.g., torsade de pointes, TdP) are rare events even in high-risk cases, it nevertheless makes sense to establish an initial QTc baseline value before the initialisation of cancer therapies. The use of medication associated with QTc prolongation should be avoided in cases of familial long QT syndrome (LQTS). Any existing QTc prolongation induced by pre-medication is particularly problematic. In addition to the typical kinase inhibitor-induced QTc prolongation, they contribute to the well-documented incidence of TdP, as in the case of vandetanib. The patient’s overall risk profile and individual QTc prolongations certainly require thorough evaluation (www.crediblemeds.org) [89].

The ECG may also be indicative of myocardial ischaemia, which may warrant additional cardiac diagnostic workup, particularly when clinicians prescribe ischaemia-inducing drugs, such as 5-FU. Moreover, if the patient has a current or prior history of atrial fibrillation, which in many cases entails the use of anticoagulants, these can further impede cancer therapy by increasing the risk of bleeding. Conversely, anticoagulants should not be unnecessarily withheld in cases of atrial fibrillation [80]. In contrast to venous thromboembolism, an increased risk of cerebral ischaemia in patients with atrial fibrillation and cancer has not been conclusively confirmed.

Patients at risk of developing cardiovascular complications should undergo echocardiography not only to detect subclinical LVEF reduction but also to allow baseline assessment of the pericardium, valves and pulmonary artery pressure for follow-up comparisons. The predictive value of a reduced LVEF for stem cell transplantation remains ambiguous. If a stem cell transplantation is planned, an LVEF of < 50% only adds one point to the *Sorror Score* for patient risk stratification [127, 128]. A mildly to moderately reduced LVEF does not appear to be an exclusion criterion for a stem cell transplant [112].

Any reduction in LVEF does, however, have predictive value for other cancers/cancer treatments [26]. This also applies to the LVEF prior to treatment with HER2 inhibitors/antibodies (after completion of anthracycline therapy) [50]. The latest American Society of Clinical Oncology (ASCO) and European Society of Cardiology (ESC) recommendations therefore classify a patient with an LVEF of less than 50–55% as a high-risk patient [5, 149].

### Cardiotoxic risk induced by prior cancer treatment

Many cancer patients undergo several potentially cardiotoxic therapies. This may occur in the context of the first oncological therapy (progression or relapse) or in the case of a secondary malignant disease. Any prior oncological therapy should therefore be considered an independent risk factor for a future potentially cardiotoxic event. This consideration is particularly applicable to anthracyclines and radiotherapy [4]. Intensified follow-up care of cancer patients is particularly important after radiotherapy of > 30 Gy, anthracycline therapy of more than 250 mg/m^2^ or after combined therapeutic approaches. Consequently, if these patients undergo additional radio- or chemotherapy they are at high risk and require thorough cardiac monitoring and surveillance.

The relevance of new imaging techniques (strain rate echocardiography, cardiac MRI, nuclear imaging) as well as genetic factors and novel biomarkers are therefore expected to increase. Current evidence relating to genetic factors mainly focuses on anthracycline toxicity [11, 57] and has not yet found its way into routine clinical practice. The importance of tumour-related cardiodepressive factors beyond lifestyle changes in cancer patients (e.g., inactivity) or risk factors such as clonal haematopoiesis still remains to be elucidated.

**Conclusions:**

- Before scheduled oncological therapy, cardiovascular risk factors and the prior history of potentially cardiotoxic therapies are decisive for individual risk assessment.

Three specific patient groups require close cardiological monitoring during oncological treatment:

- Patients with cardiovascular risk factors. These patients have an increased risk of cardiovascular side effects associated with cancer therapy.
- Patients who have undergone prior systemic therapy or radiotherapy of the chest are at risk of developing cardiotoxicity.
- All patients who undergo a therapy with known potential cardiotoxicity require an accurate baseline cardiac examination and close surveillance.

## Radiotherapy

Radiotherapy is a key component of a multimodal therapy approach for many types of cancers. It is used for local tumour control, as palliative therapy for metastatic cancer, as neo-adjuvant and adjuvant therapy in a curative approach, and as definitive treatment of localised cancers. Radiotherapy is performed within one year of diagnosis in 35% of all cancer patients [87, 141]. Cardiac radiation exposure primarily results from the irradiation of a mediastinal lymphoma, a central bronchial carcinoma, or a left-sided breast carcinoma [9, 141, 149].

Radiotherapy-associated cardiovascular complications manifest acutely but also chronically 20 years or more after exposure to radiation. Radiotherapy-associated pericarditis is an acute complication following radiotherapy [141]. The acute form was considered the most common cardiac complication after radiotherapy, but its incidence has decreased as a result of lowering the single cardiac dose and through technological advances [49]. Acute radiotherapy-associated pericarditis is characterised by a distinct pericardial immune cell infiltration that often progresses to an exsudative form. Chronic radiotherapy-associated pericarditis can manifest with variable frequency and appears only with delay after radiotherapy. Twenty percent of patients with chronic pericarditis develop a clinically relevant cardiac constriction. The cumulative cardiac radiation dose is considered to be a major risk factor; at a dose of 40 Gy, the incidence of manifest pericarditis is 5% within 5 years from irradiation [29, 141]. Pericardial effusions within one year after treatment are detected in 31.4% of radiation patients. Diagnostics primarily comprise echocardiography and cardiac catheterisation. Total pericardiectomy is the treatment of choice for constrictive pericarditis [141].

Typical chronic complications associated with radiotherapy include CAD, calcific valvular lesions and myocardial fibrosis with diastolic and, less frequently, systolic dysfunction [141, 149]. Risk factors are cumulative radiation dose, concomitant anthracycline chemotherapy, a high cardiovascular risk profile and pre-existing cardiovascular disease [58, 60, 141, 149]. Patients who survived childhood cancers represent a unique high-risk group. The relative risk of severe cardiac disease at the age of 40 is 1.9 at a cardiac radiation dose of 1–5 Gy and increases to 19.5–75.2 at a dose > 15 Gy [53].

CAD is the most frequent cardiovascular complication after radiotherapy for breast cancer. Breast cancer patients also have an increased risk of developing CAD more than 20 years after radiotherapy [32, 138]. The individual risk increases by 4.1–7.4% per 1 Gy of cardiac radiation dose administered [32, 138, 141]. Which coronary arteries are affected is dependent on the nature of the radiation fields; the irradiation of a left-sided breast carcinoma is associated with radiation exposure of the left anterior descending artery (LAD), whereas mediastinal irradiation leads to the exposure of the left main, the circumflex artery (CX) and the right coronary artery (RCA). Ostial lesions are frequent following radiotherapy [149].

Mediastinal radiotherapy of Hodgkin's lymphoma is characterised by a higher level of heart valve radiation exposure and an increased risk of valvular disease, with a latency of more than 20 years [138, 141]. At a cumulative radiation dose of > 30 Gy in particular, the relative risk increases exponentially, rising to 24.3% at a dose of > 40 Gy [30, 138]. Following chest irradiation with or without concomitant chemotherapy, valvular disease was the second most common complication in long-term survivors next to heart failure [53]. Data from the first long-term studies demonstrate an association between a lower chest radiation dose and a reduction in cardiac mortality [141].

### Management of patients after radiotherapy

A number of factors impede the diagnosis and treatment of cardiovascular complications after radiotherapy; CAD due to radiotherapy can cause atypical or attenuated symptoms as a result of radiation-induced neuropathy [73]. The complication rate of interventional and especially surgical treatments is increased by proximal lesions, mediastinal adhesions and the underlying tumour disease. Therefore, early diagnosis and treatment of radiotherapy-associated cardiovascular damage are particularly relevant.

Even before initiating chest radiotherapy, patients with an increased cardiovascular risk profile or receiving concomitant cardiotoxic chemotherapy (particularly with anthracyclines) should undergo a risk assessment. Thus, more advanced diagnostic approaches and interdisciplinary, risk-adapted therapy planning can be carried out before initiating radiotherapy [144].

**Fig. 2:**
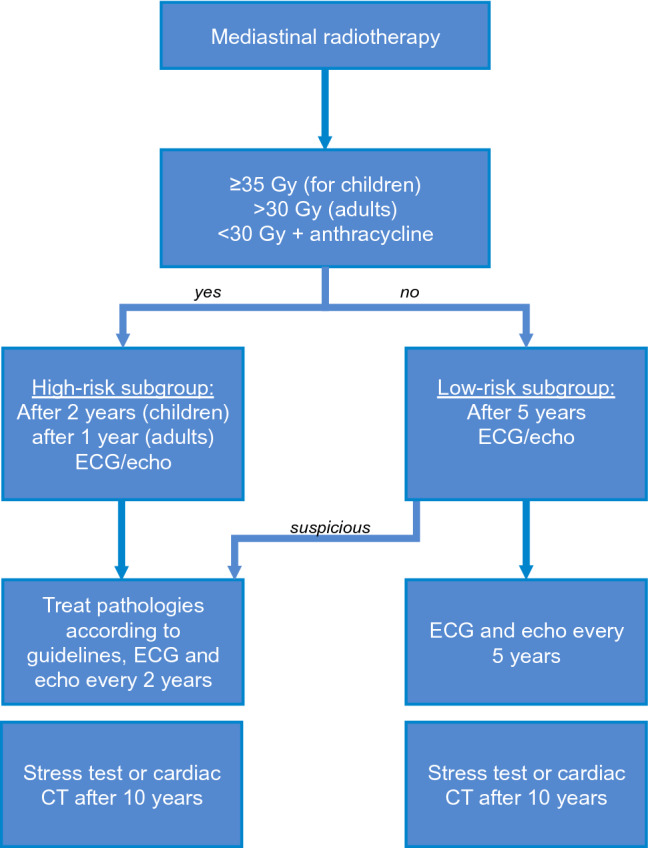
Algorithm for cardiological follow-up after mediastinal irradiation. *Gy* Gray, *ECG* electrocardiogram,* echo* echocardiography, *CT* computed tomography

To monitor cardiovascular complications associated with radiotherapy, an onco-cardiological assessment that includes an ECG and echocardiography should be carried out at intervals of 2–5 years, depending on the individual risk level, starting 5 years after irradiation exposure [21, 73, 141]. A cardiac assessment should be performed early (2 years after treatment for children and 1 year after radiotherapy for adults) in patients after receiving a ≥ 35 Gy radiotherapy dose during childhood, after a radiation dose > 30 Gy or after concomitant anthracycline therapy (Fig. 2) [4]. If the quality of echocardiography is impaired, cardiac MRI should be considered [8]. Non-invasive CAD diagnostics can be performed by coronary CT or by a stress test for ischemia 10 years after exposure to radiation [21].

## Conventional chemotherapy

Conventional cytotoxic chemotherapy can lead to a broad spectrum of cardiovascular side effects, and this is particularly true for the anthracycline class (including doxorubicin, epirubicin, daunorubicin, idarubicin, mitoxantrone), which entails a considerable risk of myocardial damage. Anthracyclines are extensively used in the treatment of acute leukaemias, breast carcinomas, sarcomas, and malignant lymphomas. Myocardial damage is caused by reactive oxygen species (ROS), the inhibition of topoisomerase IIβ, and interference with calcium channels. These changes can cause LV dysfunction, which can lead to heart failure and is an independent predictor of increased mortality. The risk of anthracycline-associated cardiotoxicity is a function of the cumulative dose, the maximum single dose and the patient's risk factors, and it is increased by concomitant thoracic irradiation or treatment with HER2 inhibitors [141]. The risk of LV dysfunction is 5% for treatment with doxorubicin at a cumulative dose of 400 mg/m^2^ and rises to 26% for a cumulative dose of 550 mg/m^2^ [149]. Doxorubicin is best evaluated as an essential chemotherapy standard for breast cancer (AC = Adriamycin^R^ + cyclophosphamide) and high-grade malignant lymphomas (CHOP = cyclophosphamide, vincristine, doxorubicin and vincristine). The doxorubicin threshold dose of 400 mg/m^2^ is reached when clinicians administer typical single doses of 50–60 mg/m^2^ for 4–6 cycles as part of adjuvant or curative chemotherapy. Below this threshold, the 5–10-year maximum likelihood of cardiomyopathy incidence is 5%, which is considered negligible. If this dose threshold is exceeded, the number of patients who develop LV dysfunctions increases exponentially, which means that additional re-exposures should be avoided and liposomal anthracyclines should be applied when possible.

Based on an assessment of the individual risk of developing anthracycline-associated cardiotoxicity, monitoring is then carried out by clinical follow-ups, ECG and echocardiography, if necessary, with 3D-LVEF and a strain analysis [141]. The assessment of cardiac troponin levels shortly after administration of therapy is highly predictive [92]. In patients at high risk for anthracycline-associated cardiotoxicity, patients whose troponin level increase as a result of therapy, and patients with an existing LV dysfunction, therapy with an angiotensin converting enzyme (ACE) inhibitor/angiotension 1 (AT1) receptor antagonist and/or a β-blocker can be used for either preventive or therapeutic purposes. To reduce anthracycline-associated cardiotoxicity, therapy with the iron chelator dexrazoxane, the administration of an alternative anthracycline formulation (pegylated and/or liposomal anthracyclines) or a switch to a non-anthracycline therapy are all acceptable approaches [21, 141].

Alkylating antineoplastic agents (cyclophosphamide, ifosfamide) are used for the therapy of malignant haematological neoplasias, solid tumours and autoimmune diseases and are associated with cardiovascular complications, particularly at the high doses given prior to bone marrow transplantation. Cyclophosphamide-associated heart failure (incidence 5–19%) typically develops within a few days after the application of treatment. Lipid peroxidation, mitochondrial dysfunction and the generation of ROS are presumed to contribute to the pathological mechanisms. Preventive therapy with ACE inhibitors/AT1 receptor antagonists and/or a β-blocker has not been sufficiently evaluated. Cyclophosphamide is used in doses between 600 and 2000 mg/m^2^ and is considered relatively safe; however, at higher doses, it can result in the cardiac damage as outlined above.

Fluoropyrimidines (5-FU) can induce coronary artery vasospasm and lead to transient angina pectoris or acute myocardial infarction. Vasospasm typically occurs in the early phase of therapy. The risk depends on the type of application, dose and individual predisposition but can be up to 10%. Calcium antagonists and nitrates have been used successfully for the treatment of coronary artery vasospasm with fluoropyrimidines [21, 92, 141]. Platinum derivatives (cisplatin, carboplatin), used in therapy, e.g., of colorectal carcinomas and testicular tumours, can also cause myocardial ischaemia due to a pro-coagulant effect and the induction of endothelial dysfunction by arterial thrombosis/thromboembolism. In addition, patients have an increased risk of developing CAD within 20 years after platinum-based chemotherapy [149].

## Immune checkpoint inhibitor-induced myocarditis

In 2018 alone, over 2250 active clinical trials evaluated the use of immune checkpoint inhibitors (ICIs) in malignant diseases. This novel class of drugs is often associated with autoimmune-mediated side effects [39, 85, 96, 97, 120]. ICI-induced myocarditis is associated with a relatively high mortality rate of 43–46% and, depending on the drug used, occurs at a frequency of 1–2% [85]. How high-risk patients can be identified, this remains unclear [16].

### Clinical symptoms

Clinical manifestations of ICI-induced myocarditis range from isolated increases in cardiac biomarkers (increase in troponin, NT-proBNP), arrhythmias and secondary signs of heart failure to fatal events [85]. Typical or atypical angina pectoris occurs in up to 37% of patients [85].

Most cases (up to 81%) of ICI-induced myocarditis occur in the first three months following the start of treatment [85]. Very few specific risk factors for ICI-induced myocarditis have been identified to date; combinational therapy consisting of different ICIs (CTLA-4 inhibitor + PD-1 inhibitor) increases the risk of developing ICI-induced myocarditis [85]. Moreover, thymoma patients appear to have an increased risk of ICI-induced myocarditis.

ICI-induced myocarditis patients are also prone to myositis (23–30%), myasthenia-like syndrome, diplopia (up to 6%) and reduced diaphragmatic function [63, 85, 97, 120]. Myocarditis can be detected in 3–10% [136] of patients with ICI-associated myasthenia and myositis in 10–15% [3]. Patients with concomitant myasthenia symptoms and myocarditis have a high mortality rate [136].

### ECG changes

**Fig. 3:**
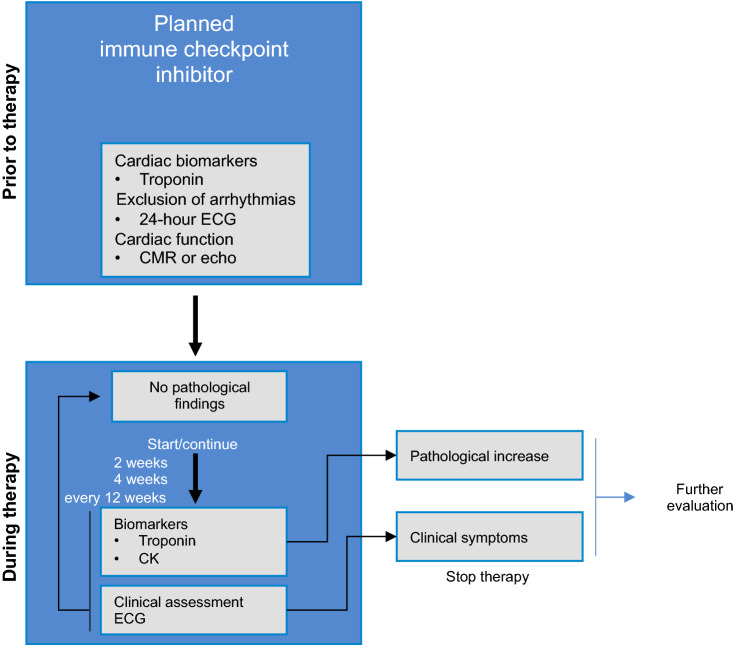
Outline of recommended baseline and follow-up examinations for immune checkpoint inhibitor therapy. Given the frequent association with myositis, it makes sense to measure troponin and creatinine kinase (CK) in parallel. *CMR* cardiac magnetic resonance imaging, *ECG* electrocardiogram, *echo* echocardiography

ECG changes are common in ICI-induced myocarditis. They range from ST elevation, ventricular arrhythmias and conduction disorders with complete AV blocks. Most patients exhibit an abnormal ECG, which appears to be one of the most reliable indicators of ICI-induced myocarditis [39, 85]. Arrhythmias are also strongly associated with ICI-induced myocarditis and are often responsible for fatal outcomes [39, 64, 85, 119, 120]. Atrial fibrillations, high-grade AV blocks, ventricular fibrillations and asystole have also been reported [96]. If ECG changes occur during ICI treatment, additional diagnostics including the measurements of cardiac biomarkers should be performed (Fig. 3), and ECG monitoring should be considered.

### Biomarkers

More than 90% of patients suggested to have ICI-induced myocarditis show increased troponin levels [96]. Although there is currently no definitive threshold for the diagnosis of ICI-induced myocarditis, higher troponin levels are associated with a greater incidence of fatal events [85]. Based on the ESC guidelines for the diagnosis and therapy of STEMI/NSTEMI, an ischaemic aetiology must first be ruled out and any other potential underlying cause of the increase in troponin must be excluded [2]. NT-proBNP is a well-established prognostic marker in heart failure patients. NT-proBNP can provide a valuable follow-up parameter, particularly in patients with severely reduced LVEF and ICI-induced myocarditis.

### Cardiac imaging

A reduced LVEF is observed only in approximately 50% of ICI-induced myocarditis cases [39, 85]. However, a reduced LVEF negatively affects outcome and is sometimes associated with considerable complications. To assess changes over time, echocardiography should be performed before initiating ICI therapy.

Cardiac MRI can confirm suspected ICI-induced myocarditis by non-ischaemic induced late gadolinium enhancement (LGE). LGE can be detected in 26–36% of patients with ICI-induced myocarditis [85]. In addition to LGE, CMR provides data on LVEF and should be considered in addition to echocardiography in patients with suspected ICI-induced myocarditis.

### Diagnostics for suspected ICI-induced myocarditis

Standard diagnostic procedures include cardiac biomarkers, an ECG and echocardiography, and, if the suspicion still remains unresolved, a cardiac MRI. Any concomitant myasthenia symptoms should also be evaluated. A significant coronary obstruction must also be excluded. A myocardial biopsy is the gold standard for the diagnosis of myocarditis and should be performed in any suspected cases. ICIs typically result in lymphocytic infiltration [64]. For additional risk stratification, a 24-h ECG should be initiated [84]. The current diagnostic criteria are summarised in Table 1.

**Table 1:** Diagnostic criteria for ICI-induced myocarditis according to Hu et al. [59]

	Histopathology	CMR	Echocardiogram with new WMA	Increased cardiac biomarkers relative to previous values
Definitive	Pathology results conclusive	CMR + M syndrome + increased cardiac biomarkers or ECG changes	WMA + M syndrome + increased cardiac biomarkers + ECG changes + absence of obstructive coronary heart disease	
Probable		CMR without M syndrome without increased cardiac biomarkers and without ECG changes	WMA + M syndrome + increased cardiac biomarkers or ECG changes	
		Inconclusive CMR findings + M syndrome or increased cardiac biomarkers or ECG changes		
Possible		Ambiguous CMR findings without M syndrome without increased cardiac biomarkers without ECG changes	WMA + M syndrome or ECG changes	Increased cardiac biomarkers + M syndrome or ECG changes

*M syndrome* myasthenia-like syndrome, *CMR* cardiac magnetic resonance imaging, *ECG* electrocardiogram, *WMA* wall motion abnormalities

### Supplementary diagnostics

Anti-acetylcholine receptor antibodies should be evaluated in patients with myasthenia syndrome to enable the consideration of additional therapeutic strategies (e.g., plasmapheresis, pyridostigmine therapy). Additional examinations (peripheral CD4/CD8 ratio, 18F-FDG PET/CT scan) may provide diagnostic confirmation in isolated cases. A skeletal muscle biopsy should be considered in patients with increased CK or with clinical signs of myositis and suspected ICI-induced myocarditis if myocardial biopsy is not feasible.

Even with a good LVEF or in the presence of significant CAD, a myocardial biopsy should be considered if ICI-induced myocarditis is suspected [39, 85]. More specifically, patients with clinical symptoms or increased CK, ECG changes, malignant arrhythmias, non-typical LGE on CMR or a reduced LVEF that is not satisfactorily accounted for by CAD require a myocardial biopsy.

### Fundamentals of ICI-induced myocarditis therapy

The current ICI-induced myocarditis therapy is primarily based on the administration of weight-adjusted steroids and supportive therapy [59] with 1–2 mg/kg prednisolone orally or by i.v. or 500–1000 mg methylprednisolone orally or by i.v. for poor responders [13]. The steroid dose should be slowly reduced over 4–6 weeks [84]. In the absence of response, when complications (high-grade arrhythmia, haemodynamic instability or deterioration of the LVEF) emerge, additional immunosuppression or plasmapheresis is recommended, especially because of the long half-lives of some drugs (Table 2). Tacrolimus, infliximab or mycophenolate are currently suggested for additional immunosuppressive therapy [59]. Furthermore, particular high-risk patients (those with haemodynamic instability, increased frequency of arrhythmias) could benefit from targeted antibody treatment with the CTLA-4 fusion protein abatacept [119]. In severe cases, an individual strategy needs to be established due to the deficient state of current research. Due to their high mortality rate, it is absolutely imperative that these patients receive intensive medical care. No further ICI therapy should be undertaken until myocarditis fully resolves. Manifest heart failure should be treated according to the current heart failure guidelines.

**Table 2:** Target structure and half-life of immune checkpoint inhibitors, modified according to [20]

	Target structure	Half-life
Ipilimumab	CTLA-4	15 days
Atezolizumab	PD-L1	27 days
Avelumab		6.1 days
Durvalumab		21 days
Nivolumab	PD-1	25 days
Pembrolizumab		27.3 days
Cemiplimab		19 days

*CTLA-4* cytotoxic t-lymphocyte-associated-protein 4, *PD-L1* programmed cell death-1 ligand 1, *PD-1* programmed cell death-1

### Basic diagnostics and monitoring of ICI therapy patients

At present, it is not known which diagnostics are absolutely necessary before ICI therapy is initiated. Due to the potential severity of myocarditis, we recommend the evaluation of cardiac biomarkers as well as the performance of echocardiography and an ECG to provide comparative results in the event of suspected ICI-induced myocarditis (Fig. 3).

ECG monitoring is recommended during therapy, particularly during the first 12 weeks due to the increased incidence of cardiac symptoms during this period. If clinical symptoms or pathological findings become apparent, further diagnostics should be performed immediately if ICI-induced myocarditis is suspected.

## Targeted therapies: conventional antibodies and ‘small-molecule’ tyrosine kinase inhibitors

Targeted therapeutics are revolutionising oncology due to their high degree of effectiveness against tumours coupled with their significantly reduced non-specific cytotoxicity. Nevertheless, targeted oncological therapeutics can also produce side effects, especially if other signalling pathways, so-called off-targets, are affected. This is particularly significant for organs and cell types that regenerate poorly, such as the heart and blood vessels. Cardiovascular side effects of current targeted oncological therapeutic agents, which are classified as frequent or very frequent according to the technical information, are listed in Table 3.

**Table 3:** Oncological indication, cardiovascular side effects and monitoring of therapy (based on technical information: often 1–10% and in bold typing very often >10%)

	Indication	Peri-/myocardial disease	Arrhythmias	Vascular diseases	Monitoring
Small-molecule tyrosine kinase inhibitors					
*BCR-ABL*					
Imatinib (PDGFR, KIT)	CML, ALL, gastrointestinal stromal tumours, chronic eosinophilic leukaemia	–	–	–	–
Dasatinib (Src)	CML, ALL	HF, pericardial effusion	QTc prolongation	HTN, PAH	ECG
Nilotinib (KIT, PDGFR)	CML	–	QTc prolongation, AF, AV block	ATE, HTN	Lipids, ECG
Bosutinib (Src)	CML	Pericardial effusion	QTc prolongation	HTN	ECG
Ponatinib (VEGFR, PDGFR, Src)	CML, ALL	HF, pericardial effusion	AF	**HTN**, VTE, ATE	BP
*BRAF*					
Vemurafenib	Melanoma, hairy cell leukaemia, multiple myeloma	–	QTc prolongation	Vasculitis	ECG, Electrolytes
Dabrafenib	Melanoma, NSCLC	LV dysfunction	–	**HTN**	
Encorafenib	Melanoma	LV dysfunction	SVT	**HTN**, VTE	TTE, ECG, Electrolytes
*MEK*					
Trametinib	Melanoma, NSCLC	LV dysfunction	Bradycardia	**HTN**	TTE, BP
Cobimetinib	Melanoma	LV dysfunction	–	**HTN**	TTE
Binimetinib	Melanoma	LV dysfunction	–	**HTN**, VTE	BP, TTE
*ALK, MET*					
Crizotinib	NSCLC	HF	**Bradycardia,** QTc prolongation	–	ECG, Electrolytes, BP
Alectinib	NSCLC	–	Bradycardia	–	
Brigatinib	NSCLC	–	Tachycardia, bradycardia, QTc prolongation	**HTN**	HR/BP
Ceritinib	NSCLC	Pericarditis	Bradycardia, QTc prolongation	–	ECG
*Bruton*					
Ibrutinib	CLL, mantle cell lymphoma, Waldenström's macroglobulinaemia	–	AF, ventr. tachyarrhythmias	–	ECG
Acalabrutinib	Lymphoplasmacytic lymphoma, mantle cell lymphoma, CLL	–	–	–	–
*EGFR*					
Erlotinib	NSCLC, pancreatic cancer	–	–	–	–
Gefitinib	NSCLC	–	–	–	–
Lapatinib (HER2)	Breast Ca.	LV dysfunction	–	–	TTE, ECG
Afatinib	NSCLC	–	–	–	–
Osimertinib	NSCLC	–	–	–	–
Neratinib (HER2)	Breast Ca.	–	–	–	–
*Multi-Target*					
Sorafenib (RAF-1/B-RAF, VEGFR2, PDGFR)	HCC, RCC, follicular thyroid Ca.	HF	–	MI, **HTN**	BP
Sunitinib (VEGFR, PDGFR, KIT)	GIST, RCC, neuroendocrine pancreatic tumour	LV dysfunction	–	Ischaemia, **HTN**, VTE	BP
Pazopanib (VEGFR, PDGFR, KIT)	RCC, soft-tissue sarcoma	LV dysfunction	–	**HTN**, VTE	BP, ECG, Electrolytes
Vandetanib (VEGFR, EGFR)	Medullary thyroid Ca.	–	**QTc prolongation**	**HTN**	ECG, Electrolytes, BP
Lenvatinib (VEGF, FGFR, PDGF, KIT, RET)	HCC, RCC, follicular thyroid Ca.	HF	QTc prolongation	**HTN**, MI, VTE	BP, ECG, Electrolytes
Regorafenib (VEGFR)	Colorectal, GIST, HCC	–	–	**HTN**	BP
Vandetanib (VEGFR, EGFR)	RCC	HF	–	**HTN**, VTE, MI	BP
Nintedanib (VEGFR, FGFR, PDGFR)	NSCLC	**–**	**–**	**–**	**–**
Cabozantinib (VEGFR, MET, RET)	RCC, HCC, medullary thyroid Ca.	–	–	**HTN, VTE, ATE**	BP, ECG, Electrolytes
Antibodies					
*HER2*					
Pertuzumab	Breast Ca.	LV dysfunction	–	–	TTE, troponin
Trastuzumab	Breast Ca., gastric Ca.	**LV dysfunction/**HF	–	**–**	ECG, TTE, troponin
Ado-trastuzumab emtansine	Breast Ca.	LV dysfunction	–	**–**	TTE, troponin
*VEGF*					
Bevacizumab	Colon Ca., breast Ca., NSCLC, RCC, ovarian Ca., cervical Ca.	HF	SVT	**HTN, VTE, ATE**	BP
Ramucirumab	Gastric, colorectal, NSCLC	**–**	–	**HTN**	BP
Aflibercept	Colorectal	**–**	–	**HTN,** VTE, ATE	BP, TTE
*EGFR*					
Panitumumab	Colorectal	–	Tachycardia	VTE, HTN	
Cetuximab	Colorectal, squamous cell carcinoma of the pharynx/larynx	**–**	**–**	**–**	**–**
Necitumumab	Squamous cell Ca., NSCLC	–	–	VTE, ATE	**–**

*EGFR* epidermal growth factor receptor, *Electrolytes* electrolytes affecting the propagation of electrical impulses such as potassium, magnesium, calcium, *HER* human epidermal growth factor receptor, *HR* heart rate, *VEGFR* vascular endothelial growth factor receptor, *PDGFR* platelet-derived growth factor receptor, *Ca.* carcinoma, *NSCLC* non-small-cell lung carcinoma, *CML* chronic myeloid leukaemia, *ALL* acute lymphocytic leukaemia, *HCC* hepatocellular carcinoma, *RCC* renal cell carcinoma, *TCa* thyroid carcinoma, *GIST* gastrointestinal stromal tumours, *HF* heart failure, *HTN* hypertension, *VTE* venous thromboembolic disease, *ATE* arterial thromboembolic disease, *MI* myocardial infarction, *PAH* pulmonary arterial hypertension, *BP* blood pressure, *TTE* ejection fraction by transthoracic echocardiography, *AF* atrial fibrillation, *SVT* supraventricular tachycardia, *Src* sarcoma proto-oncogene tyrosine-protein kinase, *KIT* stem cell factor receptor, *BRAF* rapidly accelerated fibrosarcoma Isoform B, *MEK* mitogen-activated protein kinase kinase, *ALK* anaplastic lymphoma kinase, *CLL* chronic lymphocytic leukaemia, *ECG* electrocardiogram, *HR* heart rate, *RAF* rapidly accelerated fibrosarcoma, *RET* tyrosine kinase receptor RET, *MET* receptor tyrosine kinase MET

^*^Applies only to instrument-based monitoring studies explicitly recommended for all patients in the technical information

### Multi-target tyrosine kinase inhibitors

The increase in blood pressure observed with many drugs is well illustrated by the on-target effect of the VEGFR signalling pathway. The extent of the role played by additional effects on vascular structure/vessel stiffness, which have been described, e.g., for sorafenib and sunitinib, is unclear. Many tyrosine kinase inhibitors exhibit multiple off-target effects owing to their non-specific binding to the ATP binding sites of other kinases. Some of the inhibited signalling pathways, such as RAF1 and KIT, regulate myocardial cell survival and repair mechanisms and have exhibited cardiotoxic effects both in cultured myocardial cells and in animal models [133]. The incidence of LV dysfunction reported for sorafenib and sunitinib varies between 1 and 9% [52, 100, 102]. Among tyrosine kinase inhibitors, vandetanib has been demonstrated to have the most pronounced effect on QTc prolongation, with dose-dependent rates ranging from 3 to 12% [45]. A statistically significant association of QTc prolongation with very rare malignant arrhythmias could not be detected with the study parameters used. However, 24–74% of routinely prescribed drugs have the potential to interact with cytochrome P450 3A4 metabolism, which can result in increased toxicity. Indeed, vandetanib has been shown to bind directly to cardiomyocyte hERG potassium channels in vitro and to inhibit potassium and sodium currents, resulting in attenuated depolarisation and delayed action potentials [75].

### BCR-ABL inhibitors

Imatinib is one of the first kinase inhibitors to selectively target BCR-ABL translocation, a fusion construct known to be the driving force underpinning chronic myeloid leukaemia. Little documented evidence of any relevant accumulation of cardiovascular side effects with imatinib exists. Next-generation BCR-ABL inhibitors are significantly more efficient but exhibit a number of cardiovascular side effects. Dasatinib has been found to cause pulmonary arterial hypertension in 3% of patients [51], which is reversible in approximately two out of three patients when the drug is withdrawn but may also result in permanent and clinically fatal outcomes [124]. This pulmonary arterial hypertension appears to be due to ‘off-target’ effects of dasatinib on Src kinases, which regulate smooth muscle cell proliferation and vasoconstriction. ROS-mediated endothelial adverse effects are also being discussed [51]. Therapeutic agents for the treatment of pulmonary arterial hypertension also appear to be effective for dasatinib-mediated cases [145]. Nilotinib has additionally been shown to cause dose- and time-dependent arterial vessel occlusion in over 10% of patients treated over a period of 6 years [71]. The underlying molecular mechanisms are still poorly understood. Due to the adverse metabolic effects associated with hyperglycaemia, hyperlipidaemia and secondary accelerated atherosclerosis, it is recommended that lipid metabolism parameters should be monitored prior to therapy and again at 3, 6 and every 12 months, and blood glucose levels should be monitored as well. In some patients, the changes occur rapidly and may be explained by experimentally detectable direct apoptotic and pro-atherogenic effects on vascular endothelial cells. Classic cardiovascular risk factors increase the risk of vascular side effects, and established cardiological risk scores such as SCORE can also be used to estimate the risk of vascular events in patients treated with nilotinib [114]. The third-generation multi-kinase inhibitor ponatinib has been shown to not only have a dose-dependent effect on hypertension caused by VEGFR inhibition but also to increase the risk of vascular thromboembolism complications leading to severe arterial thrombotic events in more than 20% of patients treated [27]. In experimental models, ponatinib triggers microangiopathies [72] and a prothrombogenic state by activating inflammatory mediators such as TNF-alpha, interferon-gamma, interleukin-6 and P-selectin [54]. Nevertheless, it is important to note that combination therapies are recommended specifically for those forms of acute lymphoblastic leukaemia that are difficult to treat. The potency of dasatinib is, for example, enhanced when used in combination with M199 (venetoclax), a selective inhibitor of the B-cell lymphoma 2 (BCL-2) protein [79], and is even further enhanced in combination with dexamethasone [122]. An urgent need exists for further explore the impact of such combination therapies on the cardiovascular system.

### BRAF and MEK inhibitors

The inhibition of the Ras-Raf MEK1-ERK1/2 pathway may result in impaired LV function, increased blood pressure and QTc prolongation. More specifically, a BRAF/MEK inhibitor combination therapy resulted in a higher incidence of LVEF reduction (8.1% versus 2% for monotherapy) and hypertension (19.5% versus 14% for monotherapy) than BRAF monotherapy [93]. In the case of cobimetinib, LVEF monitoring is recommended after 1 month and then every 3 months of therapy, and if LVEF decreases (< 40% or 40–49% and by > 10%), an interruption or discontinuation of therapy (if sustained < 40%) is indicated. The underlying mechanisms involved are still poorly understood. An effect of the BRAF-MEK pathway on the MAP kinases expressed by the cardiovasculature is suspected to mediate cardioprotective effects and activate nitric oxide production.

### ALK inhibitors

The electrophysiological effects of ALK inhibitors are mediated by direct effects on multiple cardiomyocyte ion channels [38, 150]. The main clinical picture is sinus bradycardia, which is generally mild or even asymptomatic [107] The maximum reduction in heart rate observed during alectinib and crizotinib therapy was on average between 13 and 25 bpm and occurred several weeks after the initiation of therapy. The strongest predictor for the development of sinus bradycardia under ALK inhibitors was a lower heart rate (< 70 bpm) before starting the therapy. Symptomatic bradycardia improves after the ALK inhibitor dose is reduced.

### Bruton's tyrosine kinase inhibitor

Ibrutinib has been reported to cause atrial fibrillation with an incidence of 3.3%. Real-world data even show a cumulative incidence of 7.5% [147]. This underestimation may be due to the often paroxysmal nature of atrial fibrillation, the often asymptomatic patients, and the retrospective design of the study. In animal models, ibrutinib leads to structural remodelling and a disruption of calcium homeostasis in the atrial myocardium, which may contribute to the development of atrial fibrillation [62]. Both Bruton's tyrosine kinase and the off-target Tec protein tyrosine kinase modulate the phosphoinositide 3-kinase PI3K-AKT signalling pathway, which has a major function in both cardioprotection and cardiac hypertrophy and is downregulated in patients with atrial fibrillation. The treatment of ibrutinib patients experiencing atrial fibrillation poses a tremendous challenge because ibrutinib is metabolised by cytochrome P450 3A4, which means that co-medication with verapamil or amiodarone can cause massive increases in circulating ibrutinib concentrations [43]. Conversely, blood levels of direct oral anticoagulants, particularly the thrombin inhibitor dabigatran, rise when administered in combination with ibrutinib, thereby increasing the risk of bleeding. Ibrutinib also independently affects various platelet signalling pathways, which generally increases the risk of bleeding. Therefore, the decision to initiate an anticoagulant treatment should be made on a case-by-case basis, with the individual consideration of the risk of stroke and, above all, of bleeding.

### HER2 inhibitors/EGF2-dependent therapeutics

The monoclonal antibody trastuzumab was one of the earliest targeted therapeutics in oncology and is predominantly used in the treatment of breast cancer. In contrast to most other active agents, therefore, considerable expertise has been acquired in the use of this drug, and concrete recommendations for onco-cardiological management are available. The epidermal growth factor (EGF) receptor 2 signalling pathway plays an important role in cardiomyocytes by compensating for stress or harmful stimulation by activating cardioprotective subcellular mechanisms involved in energy homeostasis, calcium regulation, inotropes and ultrastructure. Moreover, the inhibition of the HER2-dependent signalling pathway results in cardiomyocyte apoptosis in animal models [14]. The most significant clinical cardiovascular complications of EGF-dependent therapeutics, such as the antibodies trastuzumab/pertuzumab and the dual kinase inhibitor lapatinib, are LV dysfunction and clinically manifest heart failure. Trastuzumab is the lead compound of ‘type 2’- cardiotoxicity, which, in contrast to anthracycline-induced type 1 cardiotoxicity, was thought to be dose-independent, largely reversible and without any long-term effects. This historical classification is now, for the most part, considered obsolete, as the toxicity characteristics of these two drug classes overlap significantly.

In the initial trastuzumab studies, heart failure and LV dysfunction were observed in up to 30% of patients [126]. Due to improvements in the selection of patients, optimised therapy modalities and cardiac monitoring during therapy, event frequencies of less than 10% for trastuzumab [48] and even lower frequencies in the case of pertuzumab, emtansine and lapatinib are observed today [61]. The most important risk factors for trastuzumab-associated cardiotoxicity are prior or concurrent anthracycline therapy or a pre-existing reduced LVEF [104]. The recovery rate for trastuzumab-associated LV dysfunction is approximately 80% [148].

Clinical evaluation, ECG and an LVEF assessment prior to therapy and every 3 months during therapy, as well as every 6 months to 2 years after therapy, are recommended for trastuzumab and similarly for the other ERB2 inhibitors. Therapy should be suspended and re-evaluated after 3 weeks in the event of a reduction in the LVEF of 10% or more below 50%. This is broadly in line with the current position paper of the ESC, which, however, recommends the continuation of the therapy in the presence of ACE inhibitors if the LVEF drops to values between 45 and 49%. This latter recommendation has recently been supported by an observational study that found that when trastuzumab or pertuzumab therapy was applied to breast cancer patients with mild asymptomatic left ventricular dysfunction (LVEF 40–49%), who had been pre-treated with an ACE inhibitor and ß-blocker and monitored by a cardiologist, only 10% of patients developed cardiovascular events [83]. The clinical and economic effectiveness of monitoring in general and of particular monitoring intervals has not yet been evaluated prospectively.

A controversial primary preventive cardioprotective therapy is currently the subject of many discussions [10] and is recommended in the European position paper for high-risk patients who cannot be further stratified. The largest randomised study of 468 patients on trastuzumab found a significant benefit in the administration of preventive ACE inhibitors and β-blockers when compared to placebo, but only for patients who were also given anthracyclines [50].

If heart failure with LV dysfunction develops while taking trastuzumab, treatment based on the cardiological guidelines for heart failure is recommended.

### VEGFR inhibitors

The inhibition of the vascular endothelial growth factor receptor (VEGFR) signal transduction pathway exerts an antitumour activity by inhibiting angiogenesis, which may be mediated either by antibodies specific for the growth factor or its receptor or by the inhibition of the activity of downstream tyrosine kinases. The most significant cardiovascular side effect of all VEGFR inhibitors is an increase in blood pressure. Up to 80% of patients treated with VEGFR inhibitors developed dose-dependent hypertension, which was generally reversed by the discontinuation of the treatment. Although the increase in blood pressure correlates with the effectiveness of antitumour therapy, conversely, a drug-based blood pressure reduction protocol does not impair the effectiveness of the tumour therapy. The blood pressure increase is mediated by the inhibition of vasodilating factors such as nitric oxide and prostaglandins and an increase in vasoconstrictive factors such as endothelin, as well as a reduction in capillary vessel density [37]. The rapid increase in blood pressure after starting a therapy with VEGFR inhibitors and the lack of adaptive mechanisms increase the secondary risk of vascular events such as stroke or myocardial infarction. Randomised studies evaluating VEGFR inhibitors reported that LV dysfunction and heart failure occurred in 2.4% of patients [46], but the frequency observed in routine clinical practice appears to be higher [102]. In addition to the direct cardiotoxic effects of VEGF inhibitors, hypertension promotes the development of LV dysfunction, which appears to be at least partially reversible. The QTc prolongation effect varies widely between VEGFR inhibitors and may be attributed to direct effects on myocardial potassium channels [65].

Therefore, as part of the cardiovascular baseline assessment, it is important to optimise blood pressure parameters prior to starting VEGFR inhibitor treatment. The National Cancer Institute suggests a monitoring regime consisting of weekly blood pressure checks during the first cycle with checks every 2–3 weeks thereafter [129]. Hypertension is always treated in accordance with the relevant cardiological guidelines. Given that most VEGFR inhibitors interact with cytochrome P450 3A4, dihydropyridine calcium antagonists should be avoided. Diuretics should be used with caution as they pose a risk of electrolyte loss, which can promote QTc prolongation. First-line treatments include ACE inhibitors, angiotensin receptor inhibitors, β-blockers and non-dihydropyridine calcium antagonists. In treatment schedules involving intervals without therapy, attention must be paid to rebound hypotension and antihypertensive therapy should be adjusted accordingly. Currently, no evidence exists in support of routine screening for LV dysfunction. According to the technical information, a concrete monitoring recommendation for QTc currently applies only to vandetanib, where an initial ECG and ECG and electrolyte checks are recommended at 1, 3, 6 and 12 weeks after commencing therapy and then every 3 months. An initial QTc of greater than 480 ms is a contraindication for the administration of vandetanib.

## New haematological therapies

The most recent haematological therapies include immunomodulatory drugs and antibody therapies (using antibodies to CD38, CD20, CD79b, CD30, slam F7, CD22 or CD3/19) as well as Hedgehog signalling pathway inhibitors and PI3K inhibitors (Table 4). The following section addresses haematological drugs that belong to the proteasome inhibitor, HDAC inhibitor and immunomodulatory drug classes, as they have been reported to give rise to specific cardiac side effects and may provide useful guidance with respect to diagnosis and therapy. As far as the latest haematological therapies are concerned, more extensive analyses are required before their potentially cardiotoxic effects can be assessed.

**Table 4:** Overview of novel haematological drugs, indications and the most common potential cardiac side effects from a clinical/cardiological perspective

Active ingredient group	Active ingredient	Currently approved for*	Potential cardiac side effects
Proteasome inhibitors	Bortezomib	Multiple myeloma	Heart failure
	Carfilzomib	Multiple myeloma	Heart failure
HDAC inhibitors	Vorinostat	Cutaneous T-cell lymphoma, multiple myeloma	QTc prolongation
	Panobinostat	Multiple myeloma	QTc prolongation
	Romidepsin		QTc prolongation
Immunomodulatory drugs	Lenalidomide	Multiple myeloma	Arterial venous thrombosis, arterial hypertension, heart failure
	Pomalidomide	Multiple myeloma	Arterial venous thrombosis

*HDAC* histone deacetylase

^*^According to the EMA (European Medicines Agency); some approvals as an option only in second- or third-line therapy

### Proteasome inhibitors

In addition to reversible proteasome inhibitors (e.g., bortezomib), irreversible inhibitors with significantly prolonged and improved effects on the proteasome have been developed (e.g., carfilzomib) [55]. Carfilzomib has emerged as an option in the second-line treatment of multiple myeloma [36, 131]. Pivotal trials and toxicity studies have demonstrated that irreversible inhibitors virtually completely inhibit cardiomyocyte proteasome activity after only a relatively brief period of time. In addition, all doses tested in the preclinical model showed histological evidence of heart inflammation. A decrease in LVEF has already been reported in preclinical studies [103].

#### The clinical data

Irreversible proteasome inhibition is associated with an increased frequency of cardiovascular events, particularly heart failure. In the largest clinical trial to date, 22% of patients experienced cardiac side effects (arrhythmias, predominantly atrial fibrillation (13.3%); heart failure (7.2%); treatment-associated cardiomyopathy (2%); myocardial ischaemia (3%)) [125]. Arterial hypertension (5.9%), dyspnoea (4.5%) and heart failure NYHA class III or higher (4.4%) were reported in meta-analyses of the clinical phase I–III trials. Retrospective studies did not demonstrate a definite impact on mortality [22]. When considering individual patients who experienced cardiovascular events during bortezomib or carfilzomib treatment, classic cardiovascular risk factors were not found to be associated with an increased frequency of these events. This makes it difficult to formulate a reliable risk stratification at present.

Prospectively, approximately 65% of patients are expected to experience cardiovascular events (55% class III or higher) [26]. In the carfilzomib group, 41% of patients exhibited symptoms of heart failure, with 20% experiencing heart failure with NYHA class III or IV [26].

Eighty-six percent of cardiac side effects occurred during the first three months of therapy, which is consistent with the results from independent clinical trials. Increases in NT-proBNP and BNP (above a level of 125 pg/ml and 100 pg/ml, respectively) are prognostic markers of cardiac events. Prior radiotherapy or anthracycline treatment increases the risk of cardiovascular events, but reliable risk stratification with NT-proBNP or echocardiography does not appear to be feasible [31, 118].

#### Recommendation concerning cardiotoxicity

Patients with reduced LVEF or atrial fibrillation should be closely monitored. Cardiac biomarkers (particularly troponin) are likely to be predictive, and increases in their levels should also prompt a more densely meshed approach to care [91]. An assessment is performed in accordance with the ESC recommendations for the diagnosis and treatment of heart failure [111].

Since cardiovascular side effects seem to manifest more frequently during the first three months of therapy, patients at increased risk (pre-existing cardiac disease, extensive oncological therapies) should be monitored by a cardiologist within the first three months after initiating therapy.

#### Procedures for suspected cardiotoxicity

In cases of suspected carfilzomib-associated LVEF reduction, therapy should initially be interrupted, and heart failure therapy should be initiated in accordance with the ESC guidelines [111].

Thereafter, a new course of carfilzomib therapy can be attempted, if necessary, at a reduced dosage, albeit with strict surveillance of cardiac biomarkers and the LVEF [111].

### Histone deacetylase (HDAC) inhibitors

HDAC inhibitors act by binding and inhibiting the deacetylase domain of histone-modifying proteins. Currently approved drugs are pan-inhibitors, i.e., non-specific inhibitors with low affinity to specific HDACs. The pharmacological inhibition of HDACs not only induces a modification of the epigenome but also interacts with numerous non-nuclear HDAC target proteins, which means that the effects of HDAC inhibition are not exclusively limited to alterations in transcription. To date, only very few of the numerous HDAC inhibitors developed have been approved for clinical applications in oncology. Vorinostat is approved for the treatment of refractory and advanced cutaneous T-cell lymphoma and panobinostat for the treatment of refractory and/or relapsed multiple myeloma in patients who have received at least two previous therapies.

Although the initial clinical HDAC inhibitor trials reported supraventricular arrhythmias, [123, 134] no changes in LVEF or increases in cardiac biomarkers were observed.

Therefore, the currently available data suggest that the potentially pro-arrhythmogenic effects of HDAC inhibitors are the primary area of concern. Some of the early ECG changes include negative T waves and QTc prolongation [134]. Patients exhibiting QTc prolongations should be given time during therapy for parameters to normalise before continuing, and their electrolyte levels should also be monitored. Co-medication with any potentially QTc-prolonging drugs should be avoided. Preclinical data on cardiac fibrosis and left ventricular pump function point to potentially cardioprotective mechanisms [76].

### Immunomodulatory drugs

Immunomodulatory drugs are a class of drugs that act on tumour cells through several molecular mechanisms (the stimulation of T cells, the inhibition of haematopoietic cell proliferation, the inhibition of angiogenesis). The upregulation of TNF-alpha is associated not only with an altered release of endothelial messengers but also with an increased tendency to thrombosis. The two most commonly used drugs are lenalidomide and pomalidomide, which are both structurally derived from thalidomide.

Larger studies of immunomodulatory drugs predominantly note an increase in the number of thromboembolic events and conduction dysfunctions [42, 66]. The actual frequency of such events under immunomodulatory therapy is unclear and varies greatly depending on the clinical trial reports [74]. Thrombotic or thromboembolic events are to be expected as potential side effects in up to 23% of patients, particularly when co-treated with erythropoietin [35, 66]. Heart failure is reported in up to 4% and arterial hypertension in up to 6.9% of patients [74]. However, compared to patients treated with bortezomib, multiple myeloma patients treated with lenalidomide show no further increase in cardiovascular events [115]. In addition to thromboembolic events, sinus bradycardias and atrial fibrillation are also frequently reported [40]. Sinus bradycardia occurs independently of other ECG changes or the prolongation of the conduction times. If necessary, additional bradycardic drugs should be reduced or initialised only after renewed monitoring of the heart rate.

Due to the significant increase in thromboembolic complications of immunomodulatory therapy (thalidomide and lenalidomide), the prophylactic administration of platelet aggregation inhibitors (aspirin 100 mg) is recommended by the European Myeloma Network and the European Society for Medical Oncology (ESMO) for patients without risk factors for the development of thrombosis and the administration of heparin or vitamin K antagonists for patients with additional risk factors for thrombosis [95, 139]. This decision should be individually tailored to the patient (considering any potential contraindications for anticoagulation or platelet aggregation inhibition). Patients should also be informed about the clinical symptoms of a potential thrombosis and advised to limit thrombosis-promoting behaviours (e.g. smoking). The risk of thrombosis is further increased with the additional administration of erythropoietin.

### Chimeric antigen receptor (CAR) T-cell therapies

Targeted cardiac monitoring also appears to be indicated for emerging CAR T-cell therapies. Although there are no existing data from larger multi-centre surveys and the incidence of cardiac side effects in association with this therapeutic approach is not yet clearly defined, individual onco-cardiology working groups report cardiac side effects that may be potential ‘on-target/off-tumour’ effects of CAR T-cell therapy.

Analogously to well-established side effects such as cytokine release syndrome (CRS) or immune effector cell-associated neurotoxicity syndrome (ICANS), the emergence of an as yet undefined ‘immune effector cell-associated cardiotoxicity syndrome (OCACS)’ should be included in the spectrum of potential side effects.

## Childhood and adolescent cancers and their long-term sequelae

### Background

Childhood and adolescent cancers affect 16.8 per 100,000 individuals younger than 15 years of age. In Germany, this translates to between 1800 and 2000 new cases in this age group each year. Of these, the most prevalent is acute leukaemia, which affects 34% of this population, followed by brain tumours at 22% and lymphomas (12%).

Owing to the success of therapy optimisation, 83% of paediatric patients survive for at least 10 years and reach adulthood. This means that the number of long-term survivors increases by approximately 1500 patients per year. There are currently more than 30,000 adults who have survived childhood cancers in Germany alone.

Long-term toxicity has been reported in 60–70% of adolescent patients [105]. These predominantly comprise cardiomyopathies as well as hearing loss, impaired renal function, endocrine disorders and infertility, secondary malignancies and neuropsychological disorders. In addition to the risk of tumour relapse, cardiotoxicity plays an increasingly important role in prognosis. This is most likely because anthracycline is the most frequently administered noxious cardiac agent. The inclusion of the thorax in the irradiation field is also of considerable significance. Some 60% of oncology patients are treated with anthracyclines and/or radiotherapy. As newer drugs such as TKIs become increasingly prevalent in paediatrics, they also become increasingly important as candidates for adverse drug reactions.

The specificities of the paediatric population are important for adult follow-up care. This group of patients is mentioned twice in the ESC Statement [149]: first, they are included in the category of patients at increased risk of cardiovascular problems, and second, their requirement for lifelong follow-up care is noted, with the increased cardiovascular risk persisting for at least 45 years after cancer treatment [94].

### Cardiovascular burden following therapy in childhood and adolescence

Problems arising from childhood cancers are to be expected as a result of both the actual disease and the associated therapy. Anthracycline and/or thoracic radiotherapy present the greatest risks to the heart. Anthracyclines are among the drugs indicated for acute leukaemias, lymphomas and bone tumours [81].

Cardiotoxicity risk factors associated with the administration of anthracyclines during childhood are.

- A high cumulative anthracycline dose
- A high single dose
- Repeated dosage
- Pre-existing cardiac diseases
- Additional radiotherapy
- Lower age

Heart failure as a long-term side effect, is generally associated with a poor prognosis and accounts for half of paediatric tumour patient premature deaths [19]. There are also individual, genetically conditioned tolerances to particular chemotherapy drugs. Variants in the CUGBP Elav-like family member 4 (CELF4) gene, for instance, predispose the patient to cardiac damage following chemotherapy with anthracycline. Indeed, human cardiomyocytes differentiated from induced pluripotent stem cells (iPSC-CMs) isolated from different patients showed that such individual differences in chemotherapeutic tolerance could be reproduced experimentally from iPSC-CM [15]. The assessment of individual patients' risk of long-term cardiotoxic sequelae from cancer treatments also depends on individual differences in lifestyle and individual circumstances [23].

There is some evidence to suggest that a pregnancy subsequent to a childhood malignancy and chemotherapy may indeed present a risk for peripartum cardiomyopathy [109, 116].

There are some differences regarding the frequency and type of cardiological examination and follow-up care. Van der Pal et al. [143] examined 525 persons selected from a group of 601 long-term survivors using a set protocol. This examination essentially consisted of an M-mode echocardiographic measurement. It detected subclinical dysfunction in 27% of patients, defined as an LVEF < 30%. Risk factors included the anthracycline dose, additional radiotherapy and a lower patient age.

Alternative approaches such as 3D-EF, strain and the measurement of diastolic function are not yet sufficiently established in standard follow-up care. These methods in particular seem to detect cardiac dysfunction at an early-stage [8].

### Transition and aspects of lifelong follow-up care

Since cardiac problems following cancer therapy in childhood can manifest themselves decades later, it is important to ensure good documentation of previous therapy and long-term follow-up care. Here, it is important to develop a transition concept and evidence-based screening protocols [142]. Cardiological follow-up should begin 12 months after the end of therapy and should be carried out indefinitely at least every 5 years [4]. Pregnant women require special care. Cancer therapy survivors should be evaluated with a cardiac examination before and at the beginning of a pregnancy [109].

The objectives of paediatric initiatives will be to equip patients with the information they need for transition and to make suggestions for follow-up care. Prior to this, it is necessary to examine these patients by following a standard protocol.

Preventive strategies will need to be developed, and this must begin as early as the initial therapy.

In summary, a growing number of individuals afflicted by childhood tumours are expected to be affected by cardiovascular disease after years of latency. These survivors need to be provided with information about the disease as well as the type and extent of therapy.

## Survivorship programmes

Alongside cardiological examination prior to the start of a potentially cardiotoxic cancer treatment and regular check-ups during cancer therapy, long-term cardiac oncological care of long-term survivors is the third of the measures necessary to prevent or reduce cardiovascular consequences of cancer therapy. With the increasing success of cancer therapy, the group of long-term survivors who were exposed to cardiotoxic therapy procedures such as high anthracycline doses or aggressive radiation protocols in childhood or as adults is also growing [86, 149]. In the field of paediatric oncology, the German Childhood Cancer Registry (Deutsche Kinderkrebsregister—DKKR) is already tracking approximately 33,000 former child and adolescent cancer patients, now cured, as part of a nationwide, long-term observational study. There are still insufficient long-term results for most oncological and haematological therapies, which is why recommendations can be issued only for patients who have undergone radiotherapy or anthracycline treatment.

Although the development of new major cardiac dysfunctions after the end of anthracycline therapy is rare and, at least in breast cancer patients, cardiovascular mortality does not appear to be increased [33, 146], heart failure has been observed many years after exposure to anthracyclines [56, 130, 137]. Similarly, the consequences of mediastinal radiotherapy may take many years or decades to manifest and include valve damage, changes to small and large blood vessels, arrhythmias, myocardial and pericardial diseases and restrictive cardiomyopathy [34, 77]. Therefore, all patients who have potentially been exposed to cardiotoxic therapies should undergo a thorough clinical examination, including an ECG, at the end of cancer therapy and during regular follow-up visits (Tables 5, 6). The importance of monitoring cardiovascular risk factors should be emphasised, the awareness of the potential to develop secondary cardiovascular diseases should be increased, and any relevant symptoms should be explained.

**Table 5:** Cardiac monitoring in children and adolescents with a clinical cancer history, depending on the therapy previously administered

Risk group		Electrocardiogram and echocardiography	Cardiovascular risk factors
High	Doxo ≥ 250 mg/m^2^	After the completion of therapy Optional: 12 months after the end of therapy 24 months after the end of therapy 5 years after the end of therapy Every 5 years At any time if symptoms appear	Blood pressure at least annually Lipid profile and HbA1c at least every 3 years, especially after RTx Adjust modifiable risk factors: Nicotine Weight, BMI, WHR Explain individual risk profile Educate patient regarding lifestyle
	RTx ≥ 35 Gy		
	Doxo < 250 mg/m^2^ + RTx ≥ 15 Gy		
	Therapy at age < 5 years		
Moderate	Doxo < 250 mg/m^2^	After the completion of therapy Optional: 12 months after the end of therapy 5 years after the end of therapy Every 5 years At any time if symptoms appear	
	RTx ≥ 15 – < 35 Gy		
Other agents	Cisplatin	On a single basis	
	(High-dose) cyclophosphamide		
	Mitoxantrone		

*Doxo* doxorubicin, *RTx* radiotherapy, *Gy* Gray, *BMI* body mass index, *WHR* waist-to-hip-ratio

According to [4, 24]

**Table 6:**
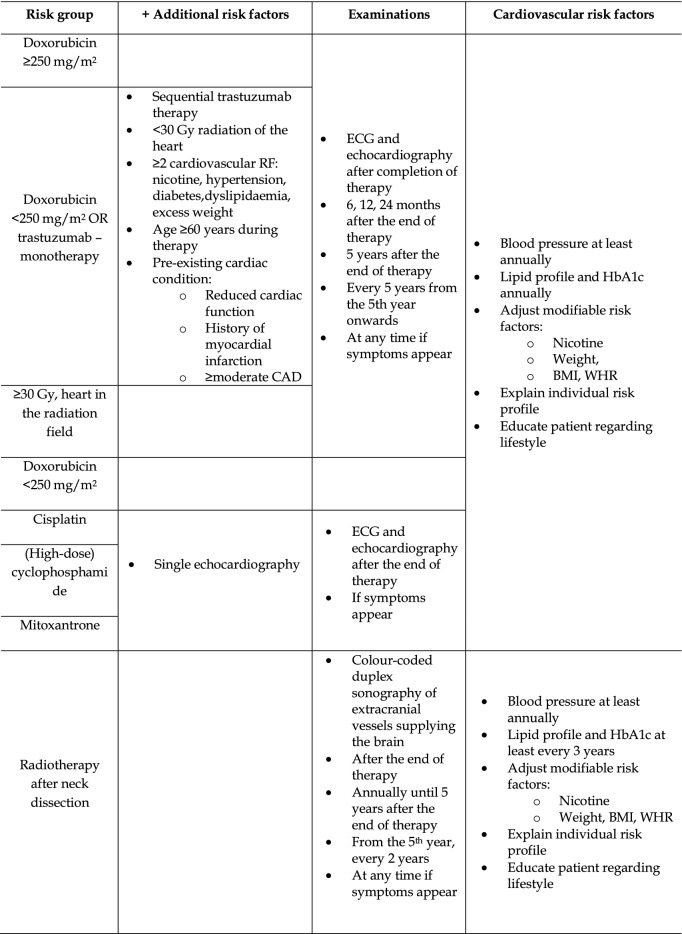
Cardiovascular monitoring in asymptomatic survivors following adult cancer therapy

*CAD* coronary artery disease, *Gy* Gray, *RF* risk factor, *WHR* waist-to-hip-ratio, *ECG* electrocardiogram, *BMI* body mass index

Modified according to [5, 19, 110]

### Follow-up observation after anthracycline therapy

Given that even low anthracycline doses are associated with heart failure or at least subclinical cardiovascular consequences and that the risk of the development of heart failure increases with other risk factors [12, 98, 144], echocardiography should also be performed in asymptomatic patients who received low anthracycline doses as part of cardiac monitoring (Tables 5, 6). Exemptions are justified only in the case of patients who have received low anthracycline doses as adults and do not present any additional risk factors (Table 6) [5]. These recommendations are in line with the recent position paper of the ‘Long-Term Observation’ working group of the German Society for Paediatric Oncology and Haematology (Gesellschaft für Pädiatrische Onkologie und Hämatologie—GPOH) [70] and the recommendations of the International Late Effects of Childhood Cancer Guideline Harmonization Group [4], who recommend that patients treated in childhood or adolescence undergo risk stratification depending on the anthracycline dose, in addition to regular echocardiographic examination and ECGs, to detect any signs of cardiomyopathy. The following online tool can also be used for risk stratification in this patient group: https://ccss.stjude.org/tools-and-documents/calculators-and-other-tools/ccss-cardiovascular-risk-calculator.html.

Programmes for long-term cardiac monitoring of patients who underwent therapy as adults have been initiated in many locations. A proposal for long-term monitoring after anthracycline or trastuzumab treatment, based on the recommendations of the American Society of Clinical Oncology, is presented in Table 6 [5]. Because cardiac complications generally occur relatively early, initial close echocardiographic monitoring should be carried out for asymptomatic patients [5, 19]. Over time, the need for echocardiography becomes less frequent, thereby minimising unnecessary concern about the effects of excessive monitoring. Since echocardiography is good at detecting diastolic dysfunction, valvular heart disease or an increase in pulmonary arterial pressure in addition to reduced LVEF, a thorough examination is essential [110, 149]. Strain analysis offers great potential as a future routine echocardiographic parameter. For the moment, however, its inclusion can be considered only on a case-by-case basis, particularly where longitudinal follow-ups are involved. As an alternative to echocardiography, a cardiac MRI can also be performed, for example, to overcome restrictive sonographic conditions. While the detection of reduced LVEF should initiate heart failure therapy even in asymptomatic patients, the significance of changes in cardiac MRI such as late gadolinium enhancement remains to be elucidated [82, 117]. The prognostic significance of biomarkers in asymptomatic long-term cancer survivors is likewise poorly understood [5]. Therefore, future studies will be required before routine screening for heart failure can be implemented. If heart failure is detected, the therapy should be based on the ESC recommendations [111], since there is currently no recommendation for a heart failure therapy specifically tailored for long-term survivors of cancer. In particular situations, such as pregnancy, all patients previously treated with anthracycline should undergo cardiac monitoring, as the increased metabolic demand may result in the progression of subclinical heart failure.

### Radiotherapy follow-up observation

While advances in radiotherapy have increased the precision with which the radiation field can be controlled, mediastinal irradiation is inevitable in some cases. If radiation-induced sclerosis or valve insufficiencies (especially of the aortic valve) are detected during regular echocardiographic monitoring, they should be reassessed annually according to a recommendation of the European Association of Cardiovascular Imaging/American Society of Echocardiography (EACVI/ASE) [68]. For patients who are asymptomatic after radiotherapy encompassing the heart, a five-year follow-up interval is sufficient according to EACVI/ASE (Tables 5, 6) [68].

Vascular changes can also occur beyond the irradiated field and often remain hidden or manifest themselves as cardiac ischaemia or stroke [25]. Long-term survivors of cervical radiotherapy in particular should undergo regular ultrasound scans of the extracranial vessels supplying the brain to detect any atherosclerotic changes, since the incidence of carotid stenosis increases by 18 to 38% after radiotherapy for head and neck tumours compared to 0 to 9.2% in patients who did not receive radiotherapy. Consequently, cardiac risk factors should be monitored more intensively, and in cases involving severe carotid stenosis, angioplasty stenting or surgery should be discussed.

## Onco-cardiology teams

### Structure

These recommendations for structuring and creating a specialised onco-cardiology group are based on data published by onco-cardiological institutions and the current ESC recommendations [41, 69, 108]. The oncology team is imbedded in the local cardiology structure and uses the latter's cardiological diagnostics. The size and constitution of the onco-cardiology teams vary according to regional needs. The size of the facility and the expected patient volume are key factors to consider, as are the established priorities in the provision of haematological and oncological care, which require corresponding adjustments to onco-cardiological care (Table 7) [28, 69, 108].

**Table 7:** Recommendation for the implementation of an onco-cardiology structure based on the current report of the Cardio-Oncology Working Group of the ESC [69]

	Basic structure/basic care	Advanced patient care/maximum care	Specialised centres
Number of patients	< 10 patients/week	> 10 patients/week	> 20 patients/week
Hospital structure	Cardiology department Oncology department General intensive care unit	Cardiology department Haematology/oncology/radiotherapy clinic Haematology department	Departments as for maximum care providers Heart failure programme Cardiology intensive care unit
Multidisciplinary teams			
Organisation	Basic onco-cardiology team or specialised cardiologist General cardiology care	Onco-cardiology team	Onco-cardiology team Cardiac rehabilitation centre Heart failure unit Valve team Research focus
Onco-cardiology outpatient clinic	Recommended	Available	Available
24/7	Recommended	Available for inpatients	Available for inpatients
Structured clinical procedures	Available	Available	Available
Cancer follow-up programme		Available	Available
Structured further education		Implemented for staff	Implemented for staff and patients
Technical requirements			
Standard echocardiography	Available	Available	Available
* Strain/3D strain*	Not mandatory	Available	Available
CMR, CT	Not mandatory	Available	Available
Laboratory tests (cardiac biomarkers)	Available	Available	Available/genetic/new biomarkers
Procedures			
Cardiac catheters/electrophysiological examinations/heart surgery/cardiac device therapy	Network with larger, regional onco-cardiology centres	Available	+ Care for terminal heart failure patients
Review of data			
Databases and research programme	Not mandatory	Strongly recommended	Implemented onco-cardiological research focus

*CMR* cardiac magnetic resonance imaging, *CT* computed tomography

### Coordination of patient routing

As different cancer treatments follow different timelines, the onco-cardiology team will need to adapt to these variations. This should be tailored to the local circumstances and can be a focal point in the outpatient and/or inpatient sectors. Particular patient groups or those with specific therapies scheduled should be seen in onco-cardiology as part of a standard procedure to be established in consultation with the treating oncologist [149].

### Basic structure

In principle, the essential requirements for clinicians for recording a medical history, carrying out a physical examination and performing an ECG should be available [69]. In terms of diagnostic imaging, the ability to perform echocardiography with strain analysis (if applicable, 3D strain analysis) should be established as a basic level of oncological patient care [110]. A diagnostic laboratory with a specific focus on determining cardiac serum parameters (troponin, BNP, NT-proBNP) should be available. More advanced cardiac diagnostics are to be performed in regional networks with larger hospitals or specialised institutions (e.g. a university onco-cardiology department). Basic onco-cardiological medical care should therefore include:

- ECG
- 24-h ECG
- echocardiography, if necessary, 3D LVEF, strain and, if necessary, 3D strain
- laboratory diagnostics, including cardiac serum parameters

### Central structures (highest level of care)

Advanced cardiac diagnostics demand the diagnostic and therapeutic resources found in a facility offering the highest level of cardiac care. In addition to basic care, this includes the following specific onco-cardiological services:

- cardiac MRI
- CT
- right heart catheterisation
- left heart catheterisation with the option of coronary intervention and myocardial biopsy
- PET-CT (previously restricted to trials)
- if necessary, facilities for diagnostic and therapeutic catheter ablation
- device therapies

To answer interdisciplinary onco-cardiological questions, regular coordination with the treating haematologist or oncologist is essential. This can be done through consultation within existing tumour boards or independently, e.g., as part of regular onco-cardiology conferences.

For the standardised follow-up patient visits (e.g., after neoadjuvant cytostatic therapy or shortly after the initialisation of immune checkpoint inhibitor therapy), internal procedures should be defined in accordance with the current position paper and the ESC position paper [149].

In the case of specialised centres, further training programmes for medical and non-medical staff (e.g., as part of a cardiac nurse course) and structured informative sessions for patients are also recommended.

If patient numbers are very high, a specific onco-cardiology consultation service can be established, which will assume an advisory function in the context of previously determined cardiac findings.

### Training

In the ‘General Cardiology’ curriculum, items relating to onco-cardiological training are listed under ‘Professional Conduct: 12′ as follows: ‘Consultation with the oncologist regarding possible constraints on the planned tumour treatment due to pre-existing heart disease as well as with regards to theoretical insights; understanding of the side effects of drugs, radiotherapy and surgical treatment on the heart.’ In terms of practical skills, level III in echocardiography is required (highest level of training).

Training in specific onco-cardiological diagnostics and therapy is given as part of cardiology further education programmes and is for the most part implemented in these programmes.

Rotations on a ward with a focus on haematology or oncology as part of a specialised onco-cardiology section/team for cardiologists who want to focus more extensively in onco-cardiology is desirable for any later.

The ability to assess cardiac risk profiles for cardiovascular diseases and to diagnose and treat cardiac complications during or after cancer treatment is essential.

This includes

- reduced LVEF
- CAD
- arrhythmias
- arterial hypertension
- PAD
- pulmonary arterial hypertension
- myocarditis
- pericarditis

The diseases listed can occur as a result of either cancer therapy or overlapping risk profiles.

It also makes sense to address the assessment of the specific cardiac risk of particular cancer therapies (e.g., the diagnosis and therapy of immune checkpoint inhibitor-induced myocarditis or the diagnosis and therapy of pulmonary hypertension related to treatments with alkylating agents) separately as part of a structured further education programme [69, 106, 113, 120].

Part of the structured further education programme should also include a basic understanding of oncology and haematology, particularly the fundamentals that underpin therapy decisions and treatment options and accurate nomenclature usage [69].

The joint position paper of the International Society of Cardiac Oncology and the Canadian Society of Onco-Cardiology calls for specialised onco-cardiology further education programmes with a range of training durations and covered contents (levels I-III) [78]. The recommendations from the German Cardiac Society will be based on the European recommendations currently being developed by the ESC.

### Selection of patients

In principle, patients selected for onco-cardiology co-supervision (Table 8) can be divided into three groups:

**Table 8:** Selection of onco-cardiology patients, adapted from the current ESC policy paper [69]

	Prior to cancer treatment	During cancer treatment	After cancer treatment
	Identification of high-cardiovascular-risk patients	Monitoring of high-cardiovascular-risk patients	Cardiological care of patients after potentially cardiotoxic therapy
	Identification of high-cardiac-risk therapies	Monitoring of high-cardiac-risk oncology patients	
		Treatment of patients with increased cardiac biomarkers or cardiac symptoms	
Responsibilities of the onco-cardiologist:	Further diagnostics and/or initiation of therapy providing advice to the oncologist	Diagnosis and therapy of pathological cardiac conditions	Initiating a cardiac therapy or initiating further cardiac diagnostics

#### Patients prior to scheduled cancer therapy

Cardiological co-supervision of oncology patients prior to cancer therapy is essential for patients presenting with a high cardiovascular risk or for a planned therapy that is associated with a high risk of cardiovascular side effects.

Therapies with an excessive risk of cardiovascular complications include

- Anthracyclines
- Immune checkpoint inhibitors
- BRAF inhibitors
- Proteasome inhibitors
- Alkylating agents
- BTK inhibitors

Depending on the results, an interdisciplinary discussion of the findings is necessary to optimise care, and, where appropriate, the treating oncologist should be consulted to reach a decision on the therapy.

#### Patients undergoing cancer therapy

An onco-cardiological coevaluation is necessary during treatment involving any of the above cancer therapies that carry an increased risk of cardiovascular side effects. Independently of this, patients undergoing cancer therapy who develop cardiac symptoms, or whose cardiac biomarkers increase, will receive (onco-) cardiological care, and the resulting cardiac findings will be evaluated in consultations.

This also applies to interdisciplinary issues related to the establishment or continuation of cancer therapy designed to optimise oncological care. An important aspect in this patient subgroup is the status of the underlying cancer (palliative, neoadjuvant, adjuvant). This also has consequences for cardiological decisions, which, for example, depend on the anticipated survival of patients and require an interdisciplinary consideration of the expected improvement in quality of life. Patients must be closely involved in the decision-making processes.

#### Patients after cancer treatment(s)

There are few available data on follow-up care after cancer treatments. In addition, most studies addressing the incidence of cardiovascular complications after cancer and cancer treatment are retrospective studies. Recommendations for the care of this patient subgroup are therefore based on the current ESC position papers [149]. It is generally the responsibility of the onco-cardiology department to inform oncology patients about any increases in cardiovascular risk following successful treatment [69]. In addition, structured follow-up programmes should be established for high-risk patients (e.g., after radiotherapy of the thorax region near the heart or anthracycline therapy).

## References

[1] Abdel-Rahman O (2019). 5-Fluorouracil-related cardiotoxicity; findings from five randomized studies of 5-fluorouracil-based regimens in metastatic colorectal cancer. Clin Colorectal Cancer.

[2] Agewall S, Giannitsis E, Jernberg T (2011). Troponin elevation in coronary vs. non-coronary disease. Eur Heart J.

[3] Anquetil C, Salem JE, Lebrun-Vignes B (2018). Immune checkpoint inhibitor-associated myositis. Circulation.

[4] Armenian SH, Hudson MM, Mulder RL (2015). Recommendations for cardiomyopathy surveillance for survivors of childhood cancer: a report from the International Late Effects of Childhood Cancer Guideline Harmonization Group. Lancet Oncol.

[5] Armenian SH, Lacchetti C, Barac A (2017). Prevention and monitoring of cardiac dysfunction in survivors of adult cancers: American society of clinical oncology clinical practice guideline. J Clin Oncol.

[6] Armenian SH, Sun CL, Francisco L (2008). Late congestive heart failure after hematopoietic cell transplantation. J Clin Oncol.

[7] Armenian SH, Xu L, Ky B (2016). Cardiovascular disease among survivors of adult-onset cancer: a community-based retrospective cohort study. J Clin Oncol.

[8] Armstrong GT, Joshi VM, Ness KK (2015). Comprehensive echocardiographic detection of treatment-related cardiac dysfunction in adult survivors of childhood cancer: results from the St. Jude lifetime cohort study. J Am Coll Cardiol.

[9] Atkins KM, Rawal B, Chaunzwa TL (2019). Cardiac radiation dose, cardiac disease, and mortality in patients with lung cancer. J Am Coll Cardiol.

[10] Barac A, Blaes A, Lynce F (2019). Lessons from primary cardiac prevention trials during trastuzumab therapy: end of one size fits all. J Am Coll Cardiol.

[11] Bhatia S (2011). Role of genetic susceptibility in development of treatment-related adverse outcomes in cancer survivors. Cancer Epidemiol Biomarkers Prev.

[12] Bowles EJ, Wellman R, Feigelson HS (2012). Risk of heart failure in breast cancer patients after anthracycline and trastuzumab treatment: a retrospective cohort study. J Natl Cancer Inst.

[13] Brahmer JR, Lacchetti C, Schneider BJ (2018). Management of immune-related adverse events in patients treated with immune checkpoint inhibitor therapy: American society of clinical oncology clinical practice guideline. J Clin Oncol.

[14] Braumann S, Peitsch WK, Pfister R (2019). Angina pectoris in a 47-year-old athletic man with psoriasis vulgaris. Internist (Berl).

[15] Burridge PW, Li YF, Matsa E (2016). Human induced pluripotent stem cell-derived cardiomyocytes recapitulate the predilection of breast cancer patients to doxorubicin-induced cardiotoxicity. Nat Med.

[16] Caforio AL, Pankuweit S, Arbustini E (2013). Current state of knowledge on aetiology, diagnosis, management, and therapy of myocarditis: a position statement of the European Society of Cardiology Working Group on myocardial and pericardial diseases. Eur Heart J.

[17] Calvillo-Arguelles O, Abdel-Qadir H, Michalowska M (2019). Cardioprotective effect of statins in patients with HER2-positive breast cancer receiving trastuzumab therapy. Can J Cardiol.

[18] Caocci G, Mulas O, Bonifacio M (2019). Recurrent arterial occlusive events in patients with chronic myeloid leukemia treated with second- and third-generation tyrosine kinase inhibitors and role of secondary prevention. Int J Cardiol.

[19] Cardinale D, Colombo A, Bacchiani G (2015). Early detection of anthracycline cardiotoxicity and improvement with heart failure therapy. Circulation.

[20] Centanni M, Moes D, Troconiz IF (2019). Clinical pharmacokinetics and pharmacodynamics of immune checkpoint inhibitors. Clin Pharmacokinet.

[21] Chang HM, Okwuosa TM, Scarabelli T (2017). Cardiovascular complications of cancer therapy: best practices in diagnosis, prevention, and management: Part 2. J Am Coll Cardiol.

[22] Chari A, Stewart AK, Russell SD (2018). Analysis of carfilzomib cardiovascular safety profile across relapsed and/or refractory multiple myeloma clinical trials. Blood Adv.

[23] Chen Y, Chow EJ, Oeffinger KC (2020). Traditional cardiovascular risk factors and individual prediction of cardiovascular events in childhood cancer survivors. J Natl Cancer Inst.

[24] Chow EJ, Baker KS, Lee SJ (2014). Influence of conventional cardiovascular risk factors and lifestyle characteristics on cardiovascular disease after hematopoietic cell transplantation. J Clin Oncol.

[25] Chow EJ, Chen Y, Hudson MM (2018). Prediction of ischemic heart disease and stroke in survivors of childhood cancer. J Clin Oncol.

[26] Cornell RF, Ky B, Weiss BM (2019). Prospective study of cardiac events during proteasome inhibitor therapy for relapsed multiple myeloma. J Clin Oncol.

[27] Cortes JE, Kim DW, Pinilla-Ibarz J (2013). A phase 2 trial of ponatinib in Philadelphia chromosome-positive leukemias. N Engl J Med.

[28] Cubbon RM, Lyon AR (2016). Cardio-oncology: concepts and practice. Indian Heart J.

[29] Cuomo JR, Sharma GK, Conger PD (2016). Novel concepts in radiation-induced cardiovascular disease. World J Cardiol.

[30] Cutter DJ, Schaapveld M, Darby SC (2015). Risk of valvular heart disease after treatment for Hodgkin lymphoma. J Natl Cancer Inst.

[31] Danhof S, Schreder M, Rasche L (2016). 'Real-life' experience of preapproval carfilzomib-based therapy in myeloma—analysis of cardiac toxicity and predisposing factors. Eur J Haematol.

[32] Darby SC, Ewertz M, Mcgale P (2013). Risk of ischemic heart disease in women after radiotherapy for breast cancer. N Engl J Med.

[33] De Azambuja E, Ameye L, Diaz M (2015). Cardiac assessment of early breast cancer patients 18 years after treatment with cyclophosphamide-, methotrexate-, fluorouracil- or epirubicin-based chemotherapy. Eur J Cancer.

[34] Desai MY, Windecker S, Lancellotti P (2019). Prevention, diagnosis, and management of radiation-associated cardiac disease: JACC scientific expert panel. J Am Coll Cardiol.

[35] Dimopoulos MA, Chen C, Spencer A (2009). Long-term follow-up on overall survival from the MM-009 and MM-010 phase III trials of lenalidomide plus dexamethasone in patients with relapsed or refractory multiple myeloma. Leukemia.

[36] Dimopoulos MA, Moreau P, Palumbo A (2016). Carfilzomib and dexamethasone versus bortezomib and dexamethasone for patients with relapsed or refractory multiple myeloma (ENDEAVOR): a randomised, phase 3, open-label, multicentre study. Lancet Oncol.

[37] Dobbin SJH, Cameron AC, Petrie MC (2018). Toxicity of cancer therapy: what the cardiologist needs to know about angiogenesis inhibitors. Heart.

[38] Doherty KR, Wappel RL, Talbert DR (2013). Multi-parameter in vitro toxicity testing of crizotinib, sunitinib, erlotinib, and nilotinib in human cardiomyocytes. Toxicol Appl Pharmacol.

[39] Escudier M, Cautela J, Malissen N (2017). Clinical features, management, and outcomes of immune checkpoint inhibitor-related cardiotoxicity. Circulation.

[40] Fahdi IE, Gaddam V, Saucedo JF (2004). Bradycardia during therapy for multiple myeloma with thalidomide. Am J Cardiol.

[41] Fiuza M, Ribeiro L, Magalhaes A (2016). Organization and implementation of a cardio-oncology program. Rev Port Cardiol.

[42] Fradley MG, Groarke JD, Laubach J (2018). Recurrent cardiotoxicity potentiated by the interaction of proteasome inhibitor and immunomodulatory therapy for the treatment of multiple myeloma. Br J Haematol.

[43] Ganatra S, Sharma A, Shah S (2018). Ibrutinib-associated atrial fibrillation. JACC Clin Electrophysiol.

[44] Gandini S, Puntoni M, Heckman-Stoddard BM (2014). Metformin and cancer risk and mortality: a systematic review and meta-analysis taking into account biases and confounders. Cancer Prev Res (Phila).

[45] Ghatalia P, Je Y, Kaymakcalan MD (2015). QTc interval prolongation with vascular endothelial growth factor receptor tyrosine kinase inhibitors. Br J Cancer.

[46] Ghatalia P, Morgan CJ, Je Y (2015). Congestive heart failure with vascular endothelial growth factor receptor tyrosine kinase inhibitors. Crit Rev Oncol Hematol.

[47] Giovannucci E, Harlan DM, Archer MC (2010). Diabetes and cancer: a consensus report. Diabetes Care.

[48] Goel S, Liu J, Guo H (2019). Decline in left ventricular ejection fraction following anthracyclines predicts trastuzumab cardiotoxicity. JACC Heart Fail.

[49] Groarke JD, Nguyen PL, Nohria A (2014). Cardiovascular complications of radiation therapy for thoracic malignancies: the role for non-invasive imaging for detection of cardiovascular disease. Eur Heart J.

[50] Guglin M, Krischer J, Tamura R (2019). Randomized trial of lisinopril versus carvedilol to prevent trastuzumab cardiotoxicity in patients with breast cancer. J Am Coll Cardiol.

[51] Guignabert C, Phan C, Seferian A (2016). Dasatinib induces lung vascular toxicity and predisposes to pulmonary hypertension. J Clin Invest.

[52] Haas NB, Manola J, Ky B (2015). Effects of adjuvant sorafenib and sunitinib on cardiac function in renal cell carcinoma patients without overt metastases: results from ASSURE, ECOG 2805. Clin Cancer Res.

[53] Haddy N, Diallo S, El-Fayech C (2016). Cardiac diseases following childhood cancer treatment. Circulation.

[54] Hamadi A, Grigg AP, Dobie G (2019). Ponatinib tyrosine kinase inhibitor induces a thromboinflammatory response. Thromb Haemost.

[55] Heckmann MB, Doroudgar S, Katus HA (2018). Cardiovascular adverse events in multiple myeloma patients. J Thorac Dis.

[56] Hequet O, Le QH, Moullet I (2004). Subclinical late cardiomyopathy after doxorubicin therapy for lymphoma in adults. J Clin Oncol.

[57] Hilfiker-Kleiner D, Ardehali H, Fischmeister R (2019). Late onset heart failure after childhood chemotherapy. Eur Heart J.

[58] Hooning MJ, Botma A, Aleman BMP (2007). Long-term risk of cardiovascular disease in 10-year survivors of breast cancer. J Natl Cancer Inst.

[59] Hu JR, Florido R, Lipson EJ (2019). Cardiovascular toxicities associated with immune checkpoint inhibitors. Cardiovasc Res.

[60] Jaworski C, Mariani JA, Wheeler G (2013). Cardiac complications of thoracic irradiation. J Am Coll Cardiol.

[61] Jerusalem G, Lancellotti P, Kim SB (2019). HER2+ breast cancer treatment and cardiotoxicity: monitoring and management. Breast Cancer Res Treat.

[62] Jiang L, Li L, Ruan Y (2019). Ibrutinib promotes atrial fibrillation by inducing structural remodeling and calcium dysregulation in the atrium. Heart Rhythm.

[63] John S, Antonia SJ, Rose TA (2017). Progressive hypoventilation due to mixed CD8(+) and CD4(+) lymphocytic polymyositis following tremelimumab–durvalumab treatment. J Immunother Cancer.

[64] Johnson DB, Balko JM, Compton ML (2016). Fulminant myocarditis with combination immune checkpoint blockade. N Engl J Med.

[65] Kloth JS, Pagani A, Verboom MC (2015). Incidence and relevance of QTc-interval prolongation caused by tyrosine kinase inhibitors. Br J Cancer.

[66] Knight R, Delap RJ, Zeldis JB (2006). Lenalidomide and venous thrombosis in multiple myeloma. N Engl J Med.

[67] Kosalka P, Johnson C, Turek M (2019). Effect of obesity, dyslipidemia, and diabetes on trastuzumab-related cardiotoxicity in breast cancer. Curr Oncol.

[68] Lancellotti P, Nkomo VT, Badano LP (2013). Expert consensus for multi-modality imaging evaluation of cardiovascular complications of radiotherapy in adults: a report from the European Association of Cardiovascular Imaging and the American Society of Echocardiography. Eur Heart J Cardiovasc Imaging.

[69] Lancellotti P, Suter TM, Lopez-Fernandez T (2019). Cardio-oncology services: rationale, organization, and implementation. Eur Heart J.

[70] Langer T, Grabow D, Kaatsch P (2018). Long-term follow-up in childhood cancer survivors—position paper 2018 of the working group "long-term follow-up" of the Society of Pediatric Oncology and Hematology (GPOH) on long-term surveillance, long-term follow-up and late effect evaluation in pediatric oncology patients. Klin Padiatr.

[71] Larson RA, Kim D-W, Issaragrilsil S (2014). Efficacy and safety of nilotinib (NIL) vs imatinib (IM) in patients (pts) with newly diagnosed chronic myeloid leukemia in chronic phase (CML-CP): long-term follow-up (f/u) of ENESTnd. Blood.

[72] Latifi Y, Moccetti F, Wu M (2019). Thrombotic microangiopathy as a cause of cardiovascular toxicity from the BCR-ABL1 tyrosine kinase inhibitor ponatinib. Blood.

[73] Lee Chuy K, Nahhas O, Dominic P (2019). Cardiovascular complications associated with mediastinal radiation. Curr Treat Options Cardiovasc Med.

[74] Lee DH, Fradley MG (2018). Cardiovascular complications of multiple myeloma treatment: evaluation, management, and prevention. Curr Treat Options Cardiovasc Med.

[75] Lee HA, Hyun SA, Byun B (2018). Electrophysiological mechanisms of vandetanib-induced cardiotoxicity: comparison of action potentials in rabbit Purkinje fibers and pluripotent stem cell-derived cardiomyocytes. PLoS ONE.

[76] Lehmann LH, Worst BC, Stanmore DA (2014). Histone deacetylase signaling in cardioprotection. Cell Mol Life Sci.

[77] Lenihan DJ, Cardinale DM (2012). Late cardiac effects of cancer treatment. J Clin Oncol.

[78] Lenihan DJ, Hartlage G, Decara J (2016). Cardio-oncology training: a proposal from the international cardioncology society and Canadian cardiac oncology network for a new multidisciplinary specialty. J Card Fail.

[79] Leonard JT, Rowley JS, Eide CA (2016). Targeting BCL-2 and ABL/LYN in Philadelphia chromosome-positive acute lymphoblastic leukemia. Sci Transl Med.

[80] Lip GY, Nieuwlaat R, Pisters R (2010). Refining clinical risk stratification for predicting stroke and thromboembolism in atrial fibrillation using a novel risk factor-based approach: the euro heart survey on atrial fibrillation. Chest.

[81] Lipshultz SE, Adams MJ, Colan SD (2013). Long-term cardiovascular toxicity in children, adolescents, and young adults who receive cancer therapy: pathophysiology, course, monitoring, management, prevention, and research directions: a scientific statement from the American Heart Association. Circulation.

[82] Lustberg MB, Reinbolt R, Addison D (2019). Early detection of anthracycline-induced cardiotoxicity in breast cancer survivors with T2 cardiac magnetic resonance. Circ Cardiovasc Imaging.

[83] Lynce F, Barac A, Geng X (2019). Prospective evaluation of the cardiac safety of HER2-targeted therapies in patients with HER2-positive breast cancer and compromised heart function: the SAFE-HEaRt study. Breast Cancer Res Treat.

[84] Lyon AR, Yousaf N, Battisti NML (2018). Immune checkpoint inhibitors and cardiovascular toxicity. Lancet Oncol.

[85] Mahmood SS, Fradley MG, Cohen JV (2018). Myocarditis in patients treated with immune checkpoint inhibitors. J Am Coll Cardiol.

[86] Mccabe MS, Bhatia S, Oeffinger KC (2013). American Society of Clinical Oncology statement: achieving high-quality cancer survivorship care. J Clin Oncol.

[87] Mclaughlin PY, Kong W, De Metz C (2018). Do radiation oncology outreach clinics affect the use of radiotherapy?. Radiother Oncol.

[88] Medeiros BC, Possick J, Fradley M (2018). Cardiovascular, pulmonary, and metabolic toxicities complicating tyrosine kinase inhibitor therapy in chronic myeloid leukemia: strategies for monitoring, detecting, and managing. Blood Rev.

[89] Meid AD, Bighelli I, Machler S (2017). Combinations of QTc-prolonging drugs: towards disentangling pharmacokinetic and pharmacodynamic effects in their potentially additive nature. Ther Adv Psychopharmacol.

[90] Meijers WC, De Boer RA (2019). Common risk factors for heart failure and cancer. Cardiovasc Res.

[91] Michel L, Mincu RI, Mahabadi AA (2020). Troponins and brain natriuretic peptides for the prediction of cardiotoxicity in cancer patients: a meta-analysis. Eur J Heart Fail.

[92] Michel L, Rassaf T, Totzeck M (2018). Biomarkers for the detection of apparent and subclinical cancer therapy-related cardiotoxicity. J Thorac Dis.

[93] Mincu RI, Mahabadi AA, Michel L (2019). Cardiovascular adverse events associated with BRAF and MEK inhibitors: a systematic review and meta-analysis. JAMA Netw Open.

[94] Moller TR, Garwicz S, Barlow L (2001). Decreasing late mortality among five-year survivors of cancer in childhood and adolescence: a population-based study in the Nordic countries. J Clin Oncol.

[95] Moreau P, San Miguel J, Sonneveld P (2017). Multiple myeloma: ESMO Clinical Practice Guidelines for diagnosis, treatment and follow-up. Ann Oncol.

[96] Moslehi JJ, Johnson DB, Sosman JA (2017). Myocarditis with immune checkpoint blockade. N Engl J Med.

[97] Moslehi JJ, Salem JE, Sosman JA (2018). Increased reporting of fatal immune checkpoint inhibitor-associated myocarditis. Lancet.

[98] Mulrooney DA, Yeazel MW, Kawashima T (2009). Cardiac outcomes in a cohort of adult survivors of childhood and adolescent cancer: retrospective analysis of the Childhood Cancer Survivor Study cohort. BMJ.

[99] Murphy KT (2016). The pathogenesis and treatment of cardiac atrophy in cancer cachexia. Am J Physiol Heart Circ Physiol.

[100] Narayan V, Keefe S, Haas N (2017). Prospective evaluation of sunitinib-induced cardiotoxicity in patients with metastatic renal cell carcinoma. Clin Cancer Res.

[101] Nelson ER, Wardell SE, Jasper JS (2013). 27-Hydroxycholesterol links hypercholesterolemia and breast cancer pathophysiology. Science.

[102] Nhola LF, Abdelmoneim SS, Villarraga HR (2019). Echocardiographic assessment for the detection of cardiotoxicity due to vascular endothelial growth factor inhibitor therapy in metastatic renal cell and colorectal cancers. J Am Soc Echocardiogr.

[103] Nowis D, Maczewski M, Mackiewicz U (2010). Cardiotoxicity of the anticancer therapeutic agent bortezomib. Am J Pathol.

[104] Nowsheen S, Aziz K, Park JY (2018). Trastuzumab in female breast cancer patients with reduced left ventricular ejection fraction. J Am Heart Assoc.

[105] Oeffinger KC, Mertens AC, Sklar CA (2006). Chronic health conditions in adult survivors of childhood cancer. N Engl J Med.

[106] Okwuosa TM, Akhter N, Williams KA (2015). Building a cardio-oncology program in a small- to medium-sized, nonprimary cancer center, academic hospital in the USA: challenges and pitfalls. Future Cardiol.

[107] Ou SH, Tang Y, Polli A (2016). Factors associated with sinus bradycardia during crizotinib treatment: a retrospective analysis of two large-scale multinational trials (PROFILE 1005 and 1007). Cancer Med.

[108] Parent S, Pituskin E, Paterson DI (2016). The cardio-oncology program: a multidisciplinary approach to the care of cancer patients with cardiovascular disease. Can J Cardiol.

[109] Pfeffer TJ, Schlothauer S, Pietzsch S (2019). Increased cancer prevalence in peripartum cardiomyopathy. JACC CardioOncol.

[110] Plana JC, Galderisi M, Barac A (2014). Expert consensus for multimodality imaging evaluation of adult patients during and after cancer therapy: a report from the American Society of Echocardiography and the European Association of Cardiovascular Imaging. J Am Soc Echocardiogr.

[111] Ponikowski P, Voors AA, Anker SD (2016). 2016 ESC Guidelines for the diagnosis and treatment of acute and chronic heart failure: the task force for the diagnosis and treatment of acute and chronic heart failure of the European Society of Cardiology (ESC) developed with the special contribution of the Heart Failure Association (HFA) of the ESC. Eur Heart J.

[112] Qazilbash MH, Amjad AI, Qureshi S (2009). Outcome of allogeneic hematopoietic stem cell transplantation in patients with low left ventricular ejection fraction. Biol Blood Marrow Transplant.

[113] Ranchoux B, Gunther S, Quarck R (2015). Chemotherapy-induced pulmonary hypertension: role of alkylating agents. Am J Pathol.

[114] Rea D, Mirault T, Raffoux E (2015). Usefulness of the 2012 European CVD risk assessment model to identify patients at high risk of cardiovascular events during nilotinib therapy in chronic myeloid leukemia. Leukemia.

[115] Reneau JC, Asante D, Van Houten H (2017). Cardiotoxicity risk with bortezomib versus lenalidomide for treatment of multiple myeloma: a propensity matched study of 1790 patients. Am J Hematol.

[116] Ricke-Hoch M, Hoes MF, Pfeffer TJ (2019). In peripartum cardiomyopathy Plasminogen Activator Inhibitor-1 is a potential new biomarker with controversial roles. Cardiovasc Res.

[117] Rodriguez-Veiga R, Igual B, Montesinos P (2017). Assessment of late cardiomyopathy by magnetic resonance imaging in patients with acute promyelocytic leukaemia treated with all-trans retinoic acid and idarubicin. Ann Hematol.

[118] Rosenthal A, Luthi J, Belohlavek M (2016). Carfilzomib and the cardiorenal system in myeloma: an endothelial effect?. Blood Cancer J.

[119] Salem JE, Allenbach Y, Vozy A (2019). Abatacept for severe immune checkpoint inhibitor-associated myocarditis. N Engl J Med.

[120] Salem JE, Manouchehri A, Moey M (2018). Cardiovascular toxicities associated with immune checkpoint inhibitors: an observational, retrospective, pharmacovigilance study. Lancet Oncol.

[121] Sara JD, Kaur J, Khodadadi R (2018). 5-fluorouracil and cardiotoxicity: a review. Ther Adv Med Oncol.

[122] Scherr M, Kirchhoff H, Battmer K (2019). Optimized induction of mitochondrial apoptosis for chemotherapy-free treatment of BCR-ABL+acute lymphoblastic leukemia. Leukemia.

[123] Shah MH, Binkley P, Chan K (2006). Cardiotoxicity of histone deacetylase inhibitor depsipeptide in patients with metastatic neuroendocrine tumors. Clin Cancer Res.

[124] Shah NP, Wallis N, Farber HW (2015). Clinical features of pulmonary arterial hypertension in patients receiving dasatinib. Am J Hematol.

[125] Siegel D, Martin T, Nooka A (2013). Integrated safety profile of single-agent carfilzomib: experience from 526 patients enrolled in 4 phase II clinical studies. Haematologica.

[126] Slamon DJ, Leyland-Jones B, Shak S (2001). Use of chemotherapy plus a monoclonal antibody against HER2 for metastatic breast cancer that overexpresses HER2. N Engl J Med.

[127] Sorror ML, Giralt S, Sandmaier BM (2007). Hematopoietic cell transplantation specific comorbidity index as an outcome predictor for patients with acute myeloid leukemia in first remission: combined FHCRC and MDACC experiences. Blood.

[128] Sorror ML, Maris MB, Storb R (2005). Hematopoietic cell transplantation (HCT)-specific comorbidity index: a new tool for risk assessment before allogeneic HCT. Blood.

[129] Steingart RM, Bakris GL, Chen HX (2012). Management of cardiac toxicity in patients receiving vascular endothelial growth factor signaling pathway inhibitors. Am Heart J.

[130] Steinherz LJ, Steinherz PG, Tan CT (1991). Cardiac toxicity 4 to 20 years after completing anthracycline therapy. JAMA.

[131] Stewart AK (2015). Carfilzomib for the treatment of patients with relapsed and/or refractory multiple myeloma. Future Oncol.

[132] Stocks T, Van Hemelrijck M, Manjer J (2012). Blood pressure and risk of cancer incidence and mortality in the Metabolic Syndrome and Cancer Project. Hypertension.

[133] Stuhlmiller TJ, Zawistowski JS, Chen X (2017). Kinome and transcriptome profiling reveal broad and distinct activities of erlotinib, sunitinib, and sorafenib in the mouse heart and suggest cardiotoxicity from combined signal transducer and activator of transcription and epidermal growth factor receptor inhibition. J Am Heart Assoc.

[134] Subramanian S, Bates SE, Wright JJ (2010). Clinical toxicities of histone deacetylase inhibitors. Pharmaceuticals.

[135] Sun H, Li T, Zhuang R (2017). Do renin-angiotensin system inhibitors influence the recurrence, metastasis, and survival in cancer patients?: Evidence from a meta-analysis including 55 studies. Medicine (Baltimore).

[136] Suzuki S, Ishikawa N, Konoeda F (2017). Nivolumab-related myasthenia gravis with myositis and myocarditis in Japan. Neurology.

[137] Swain SM, Whaley FS, Ewer MS (2003). Congestive heart failure in patients treated with doxorubicin: a retrospective analysis of three trials. Cancer.

[138] Taylor C, Correa C, Duane FK (2017). Estimating the risks of breast cancer radiotherapy: evidence from modern radiation doses to the lungs and heart and from previous randomized trials. J Clin Oncol.

[139] Terpos E, Kleber M, Engelhardt M (2015). European Myeloma Network guidelines for the management of multiple myeloma-related complications. Haematologica.

[140] Tilemann LM, Heckmann MB, Katus HA (2018). Cardio-oncology: conflicting priorities of anticancer treatment and cardiovascular outcome. Clin Res Cardiol.

[141] Totzeck M, Schuler M, Stuschke M (2019). Cardio-oncology—strategies for management of cancer-therapy related cardiovascular disease. Int J Cardiol.

[142] Trachtenberg BH, Landy DC, Franco VI (2011). Anthracycline-associated cardiotoxicity in survivors of childhood cancer. Pediatr Cardiol.

[143] Van Der Pal HJ, Van Dalen EC, Hauptmann M (2010). Cardiac function in 5-year survivors of childhood cancer: a long-term follow-up study. Arch Intern Med.

[144] Van Nimwegen FA, Schaapveld M, Janus CP (2015). Cardiovascular disease after Hodgkin lymphoma treatment: 40-year disease risk. JAMA Intern Med.

[145] Weatherald J, Chaumais MC, Savale L (2017). Long-term outcomes of dasatinib-induced pulmonary arterial hypertension: a population-based study. Eur Respir J.

[146] Weberpals J, Jansen L, Muller OJ (2018). Long-term heart-specific mortality among 347 476 breast cancer patients treated with radiotherapy or chemotherapy: a registry-based cohort study. Eur Heart J.

[147] Wiczer TE, Levine LB, Brumbaugh J (2017). Cumulative incidence, risk factors, and management of atrial fibrillation in patients receiving ibrutinib. Blood Adv.

[148] Yoon HJ, Kim KH, Kim HY (2019). Impacts of non-recovery of trastuzumab-induced cardiomyopathy on clinical outcomes in patients with breast cancer. Clin Res Cardiol.

[149] Zamorano JL, Lancellotti P, Rodriguez Munoz D (2016). 2016 ESC Position Paper on cancer treatments and cardiovascular toxicity developed under the auspices of the ESC Committee for Practice Guidelines: The Task Force for cancer treatments and cardiovascular toxicity of the European Society of Cardiology (ESC). Eur Heart J.

[150] Zhang Z, Huang TQ, Nepliouev I (2017). Crizotinib inhibits hyperpolarization-activated cyclic nucleotide-gated channel 4 activity. Cardiooncology.

